# A Genome-Wide Metabolic QTL Analysis in Europeans Implicates Two Loci Shaped by Recent Positive Selection

**DOI:** 10.1371/journal.pgen.1002270

**Published:** 2011-09-08

**Authors:** George Nicholson, Mattias Rantalainen, Jia V. Li, Anthony D. Maher, Daniel Malmodin, Kourosh R. Ahmadi, Johan H. Faber, Amy Barrett, Josine L. Min, N. William Rayner, Henrik Toft, Maria Krestyaninova, Juris Viksna, Sudeshna Guha Neogi, Marc-Emmanuel Dumas, Ugis Sarkans, The MolPAGE Consortium, Peter Donnelly, Thomas Illig, Jerzy Adamski, Karsten Suhre, Maxine Allen, Krina T. Zondervan, Tim D. Spector, Jeremy K. Nicholson, John C. Lindon, Dorrit Baunsgaard, Elaine Holmes, Mark I. McCarthy, Chris C. Holmes

**Affiliations:** 1 Department of Statistics, University of Oxford, Oxford, United Kingdom; 2 Biomolecular Medicine, Department of Surgery and Cancer, Imperial College London, London, United Kingdom; 3 Biosciences Research Division, Department of Primary Industries, Bundoora, Australia; 4 Novo Nordisk A/S, Måløv, Denmark; 5 Department of Twin Research and Genetic Epidemiology, King's College London, London, United Kingdom; 6 Oxford Centre for Diabetes, Endocrinology, and Metabolism, University of Oxford, Oxford, United Kingdom; 7 Wellcome Trust Centre for Human Genetics, University of Oxford, Oxford, United Kingdom; 8 European Bioinformatics Institute, Wellcome Trust Genome Campus, Hinxton, Cambridge, United Kingdom; 9 Institute of Mathematics and Computer Science, Riga, Latvia; 10 NIHR Cambridge Biomedical Research Centre, Institute of Metabolic Science, Addenbrooke's Hospital, Cambridge, United Kingdom; 11 Institute of Epidemiology, Helmholtz Zentrum München, Neuherberg, Germany; 12 Institute of Experimental Genetics, Genome Analysis Center, Helmholtz Zentrum München, Neuherberg, Germany; 13 Institute of Experimental Genetics, Life and Food Science Center Weihenstephan, Technische Universität München, Freising-Weihenstephan, Germany; 14 Institute of Bioinformatics and Systems Biology, Helmholtz Zentrum München, Neuherberg, Germany; 15 Faculty of Biology, Ludwig-Maximilians-Universität, Planegg-Martinsried, Germany; 16 Oxford NIHR Biomedical Research Centre, Churchill Hospital, Oxford, United Kingdom; Stanford University School of Medicine, United States of America

## Abstract

We have performed a metabolite quantitative trait locus (mQTL) study of the ^1^H nuclear magnetic resonance spectroscopy (^1^H NMR) metabolome in humans, building on recent targeted knowledge of genetic drivers of metabolic regulation. Urine and plasma samples were collected from two cohorts of individuals of European descent, with one cohort comprised of female twins donating samples longitudinally. Sample metabolite concentrations were quantified by ^1^H NMR and tested for association with genome-wide single-nucleotide polymorphisms (SNPs). Four metabolites' concentrations exhibited significant, replicable association with SNP variation (8.6×10^−11^<*p*<2.8×10^−23^). Three of these—trimethylamine, 3-amino-isobutyrate, and an *N*-acetylated compound—were measured in urine. The other—dimethylamine—was measured in plasma. Trimethylamine and dimethylamine mapped to a single genetic region (hence we report a total of three implicated genomic regions). Two of the three hit regions lie within haplotype blocks (at 2p13.1 and 10q24.2) that carry the genetic signature of strong, recent, positive selection in European populations. Genes *NAT8* and *PYROXD2*, both with relatively uncharacterized functional roles, are good candidates for mediating the corresponding mQTL associations. The study's longitudinal twin design allowed detailed variance-components analysis of the sources of population variation in metabolite levels. The mQTLs explained 40%–64% of biological population variation in the corresponding metabolites' concentrations. These effect sizes are stronger than those reported in a recent, targeted mQTL study of metabolites in serum using the targeted-metabolomics Biocrates platform. By re-analysing our plasma samples using the Biocrates platform, we replicated the mQTL findings of the previous study and discovered a previously uncharacterized yet substantial familial component of variation in metabolite levels in addition to the heritability contribution from the corresponding mQTL effects.

## Author Summary

Physiological concentrations of metabolites—small molecules involved in biochemical processes in living systems—can be measured and used to diagnose and predict disease states. A common goal is to detect and clinically exploit statistical differences in metabolite concentrations between diseased and healthy individuals. As a basis for the design and interpretation of case-control studies, it is useful to have a characterization of metabolic diversity amongst healthy individuals, some of which stems from inter-individual genetic variation. When a single genetic locus has a sufficiently strong effect on metabolism, its genomic position can be determined by collecting metabolite concentration data and genome-wide genotype data on a set of individuals and searching for associations between the two data sets—a so-called metabolite quantitative trait locus (mQTL) study. By so tracing mQTLs, we can identify the genetic drivers of metabolism, characterize how the nature or quantity of the corresponding expressed protein(s) feeds forward to influence metabolite levels, and specify disease-predictive models that incorporate mutual dependence amongst genetics, environment, and metabolism.

## Introduction

Expression quantitative trait loci (eQTL) studies have proved a powerful aid to functional genomics, with many thousand genetic loci now highlighted that affect RNA transcription levels or splicing in human tissues [1]. eQTL studies have accelerated the characterization of biological mechanisms governing gene regulation [2]–[5], and genome-wide multi-tissue maps of known eQTLs have clarified the biological basis for a proportion of disease-associated [6]–[7] and positively selected [8] loci (e.g. http://eqtl.uchicago.edu/cgi-bin/gbrowse/eqtl/). Genetic variation at eQTLs can be incorporated into network models that help define dependence between genotypes, molecular traits, environment, and physiological states [9]–[10]. The success of eQTL studies points to the potential value in applying the eQTL paradigm to other molecular traits besides mRNA transcript levels [11]–[14]. In the current study, we associate genome-wide genetic variation with concentrations of *metabolites*, small molecules involved in biochemical processes in living systems, which can be measured in samples such as biofluids and tissue extracts using ^1^H nuclear magnetic resonance spectroscopy (^1^H NMR) [15]–[17], or by the *Biocrates platform*. (For convenience, we use the term ‘Biocrates platform’ in the current paper to refer to the targeted-metabolomic platform using flow-injection tandem mass spectrometry—FIA-MS—developed by Biocrates Life Sciences [14], [18].)

Metabolites are mechanistically further removed from the genome than are mRNAs, creating an important qualitative distinction between metabolite QTL (mQTL) and eQTL studies. The mRNA-to-gene mapping is a useful property of eQTL studies, allowing the search for a *cis* eQTL of each mRNA to be focused on a relatively small, gene-centred region. Moreover, most known eQTLs are *cis*-acting single-nucleotide polymorphisms (SNPs), lying usually within tens of kb of the genes whose expression they influence [1], [5]. Whilst metabolite concentrations are influenced indirectly by mRNA and protein expression, there is not typically a one-to-one metabolite-to-gene correspondence known, or indeed expected, *a priori.* An mQTL study tests variation in each metabolite for association with genome-wide genetic variation. As such a large number of tests is performed, effect sizes must be substantially larger to be reach statistical significance. Thus, as well as being potentially rarer, mQTLs are typically more difficult to detect than eQTLs of equivalent effect size.

A number of recent studies have reported mQTLs for serum metabolite concentrations in humans [14], [19]. Illig et al. [14] genotyped 1,809 individuals of Northern European ancestry at genome-wide single-nucleotide polymorphisms (SNPs), and determined concentrations of 163 metabolites in serum samples from the same individuals, using the Biocrates platform (targeted metabolomics using FIA-MS) [18]. They went on to quantify association between each SNP and a derived set of 26,569 metabolic traits (including 163 raw metabolite concentrations and all pair-wise metabolite concentration ratios). They discovered nine significant, replicable associations between metabolite concentration ratios and SNPs. We demonstrate in the current paper that their study [14] was well powered to detect mQTLs explaining approximately 3% or more of population variation in those serum metabolites targeted by Biocrates. In the current paper the *effect size* of an mQTL is defined to be the proportion of population variation in metabolite concentration that is explained by genetic variation at the corresponding mQTL SNP.

The primary question addressed by our study is: ‘Are there ^1^H NMR-detectable metabolites in urine or plasma that are strongly influenced by common single-locus genetic variation?’ To this end, we performed an mQTL-discovery study using ^1^H NMR to analyse plasma and urine samples from multiple cohorts (see Results and *Materials and Methods*). ^1^H NMR is an untargeted, discovery-driven approach that covers many important substances involved in major biochemical functions and key intermediary processes [16]. Our study demonstrates the existence of mQTLs of larger effect size than those reported in [14] for the untargeted set of metabolites detectable by ^1^H NMR, in urine as well as plasma (urine is previously unexplored for mQTLs). The current paper's secondary aim was to provide further support for the findings of [14]. We conducted replication of the findings of [14], using the Biocrates platform to assay our set of plasma samples. We replicated additional mQTLs, and characterized the familial component of biological variation in mQTL-driven metabolite levels, augmenting the mQTL-derived heritability.

## Results

### Cohorts and data acquisition

We collected plasma and urine samples from participants across two cohorts—MolTWIN and MolOBB—as part of the MolPAGE programme. The MolTWIN cohort comprised 142 female twins of Northern European descent, who donated samples longitudinally. The MolOBB cohort comprised 69 participants in the Oxford Biobank (OBB) [20].

For all participants across both cohorts we acquired: ^1^H NMR spectra on plasma and urine samples; Biocrates-platform metabolite concentration data on plasma samples; and genome-wide SNP data (see *Materials and Methods*).

### Extraction of metabolite peaks from spectra and genome-wide scan (^1^H NMR)

Analysis of a biological sample by ^1^H NMR provides a spectrum, which is comprised of the superimposed spectral profiles of individual metabolites; a metabolite's profile is made up of peaks from each chemically distinct hydrogen atom in the corresponding molecule. The peak position of a given hydrogen on the horizontal (frequency) axis is known as a *chemical shift* and is quoted in parts per million (ppm, often termed a 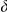 value) from that of a reference substance. The concentration of each detectable hydrogen-containing metabolite can be inferred from the area under its total specific profile, or under a specific peak if the number of protons contributing to it is known. We preprocessed spectra, and extracted a total of 526 metabolite peaks from each pair of samples, i.e. the two samples (plasma and urine) donated by a participant on a visit to the clinic. These peaks represent fewer than 526 metabolites with some redundancy (see *Materials and Methods*).

Using data from the MolTWIN cohort, each of the 526 metabolite peaks was tested for association with 2,541,644 autosomal SNPs (of which 2,245,627 were imputed and 296,017 were typed). In order to address both multiple testing and the kinship of twin pairs, we used a permutation-based procedure, constraining the genome-wide false-discovery probability to be less than 0.001 for each metabolite peak's genome-wide scan.

We detected, and then replicated, four metabolites driven largely by SNP variation (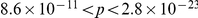), across three genomic regions, explaining between 40%–64% of biological population variation in these four metabolites' concentrations. Genetic details of the hit regions are shown in Table 1. Note that there are only three hit regions for the four metabolites because two metabolites mapped to a single, shared region. One of the mQTLs is in strong linkage disequilibrium (LD, reviewed in [21]) with SNP variation associated with renal function [22]–[23]. We found that two of the three mQTL regions exhibited genetic evidence of having experienced strong, recent positive selection in European populations (further details of these findings are presented later, in dedicated sections in Results and Discussion).

**Table 1:** Genetic details of ^1^H NMR mQTL regions.

						Allele Frequency^a^		
ID	SNP	Chr	Position^b^	Local Genes	Alleles^c^	MolTWIN	CEU^d^	YRI^e^
TMAu	rs7072216	10	100156853	*PYROXD2* (*C10orf33*)	C/T	0.35	0.25	0.86
N-ACu	rs9309473	2	73743982	*ALMS1*, *NAT8*, *TPRKB*, *DUSP11*	G/A	0.25	0.21	0.59
BAIBu	rs37369	5	35037115	*AGXT2*	T/C	0.10	0.09	0.67
DMAp	rs6584194	10	100160399	*PYROXD2* (*C10orf33*)	C/T	0.35	0.37	0.90

a Frequency of minor allele (where minor/major alleles are defined by their frequency in the Northern European HapMap-CEU population [29]).

b NCBI build 37 coordinates.

c Minor/major allele in HapMap-CEU.

d Frequency in Northern European (HapMap-CEU) population.

e Frequency in African (HapMap-YRI) population [29].

### Identification of metabolites (^1^H NMR)

We proceeded to identify as many of the mQTL-driven metabolites as possible using a combination of: the web-based human metabolome database [24], our in-house developed database, statistical total correlation analysis [25], and other literature [26]. We unambiguously identified three out of four metabolites, and partially identified the fourth. The mQTL at chromosome 10q24.2 had two associated metabolites, identified as trimethylamine in urine (TMAu), and dimethylamine in plasma (DMAp). The mQTL at 5p13.2 affects urine concentration of 3-amino-isobutyrate (a.k.a. β-amino-isobutyrate, denoted by BAIBu).

The mQTL at 2p13.1 associates with concentrations of one or more urine metabolites that we partially identified as *N*-acetylated compound(s): X.NH.CO.CH_3_, with X unknown; we denote this set of one or more metabolites as N-ACu. We were unable to annotate N-ACu unambiguously despite conducting a number of additional experiments, including: seven experiments in which we spiked candidate compounds into selected urine samples and then re-measured the ^1^H NMR spectra; solid phase extraction experiments on urine samples in which we attempted to separate out N-ACu and thus aid its identification; and 2-dimensional ^1^H-^13^C heteronuclear single quantum coherence NMR spectroscopy experiments on selected urine samples.

Table 2 summarizes these metabolite annotations. Figure S1 displays the three mQTL-driven urine metabolite peaks on the same scale, allowing visual assessment of their relative size.

**Table 2:** Annotation of mQTL-driven ^1^H NMR-detectable metabolites.

ID	Data Set	Peak ppm Interval	Metabolite	Formula
TMAu	Urine Standard 1d	(2.857 - 2.87)^a^	trimethylamine	C_3_H_9_N
N-ACu	Urine Standard 1d	(2.034 - 2.042)	*N*-acetylated compound(s)	X.NH.CO.CH_3_ b
BAIBu	Urine Standard 1d	(1.185 - 1.191)	3-amino-isobutyrate	CH_3_.CH.(CH_2_.NH_2_).COOH
DMAp	Plasma Spin-Echo	(2.7 - 2.724)	dimethylamine	(CH_3_)_2_.NH

a We observed frequency shifts in TMA peaks between the MolTWIN and MolOBB data sets, attributable to inter-study differences in experimental conditions, such as sample pH or temperature. To align the peaks across cohorts, a different peak interval was used for TMAu in the MolOBB data: (2.86 – 2.88).

b The metabolite was partially identified as an *N*-acetylated compound: X.NH.CO.CH_3_, with X unknown.

### Mixed-effects analysis of hit regions (^1^H NMR)

We went on to characterize more accurately each metabolite's associations with SNPs within 200 kb of the hit regions. We used a linear mixed-effects model to account for: the sharing of genes and environment across twins, the collection of multiple samples longitudinally from some subjects, and the technical replication of each biological sample (see *Materials and Methods*).

**Figure 1:**
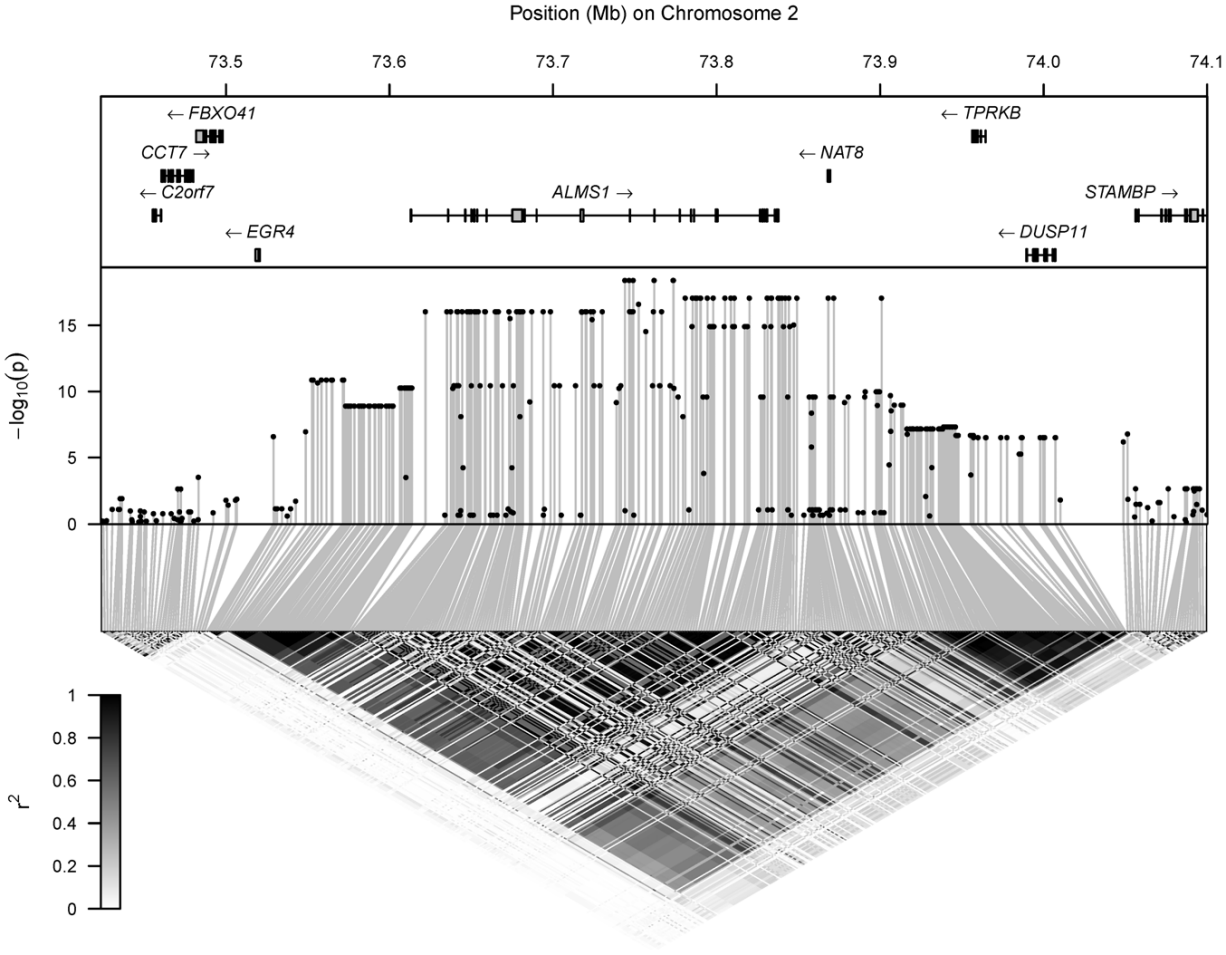
Hit region for N-ACu. Top: location of genes, with rectangles denoting the position of exons. Middle: log-transformed p-values (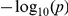) for the test of association of the metabolite's concentration with each SNP in the region. Bottom: LD between each pair of SNPs in the region, with the colour scale for 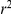 superimposed.

**Figure 2:**
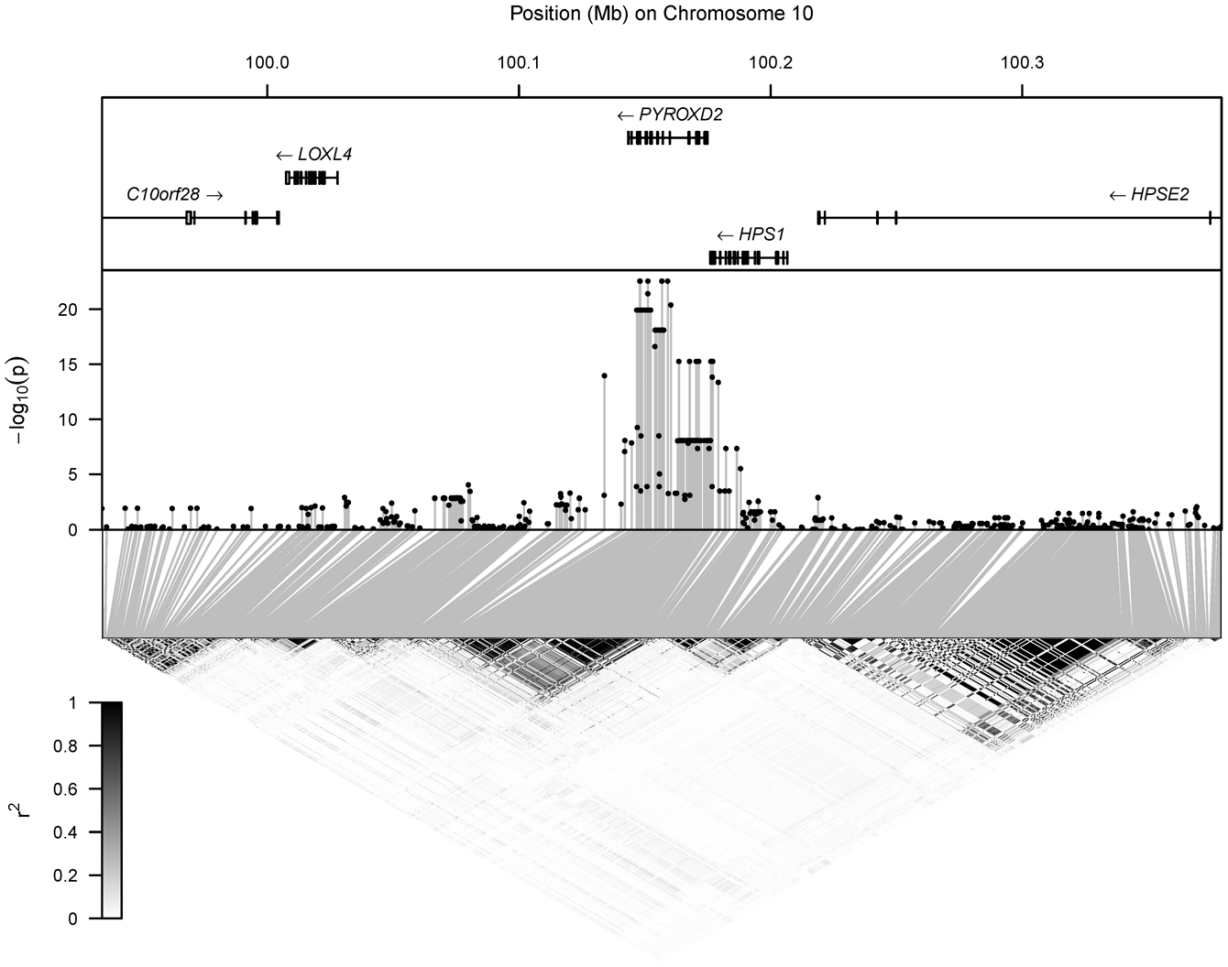
Hit region for TMAu. Top: location of genes, with rectangles denoting the position of exons. Middle: log-transformed p-values (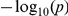) for the test of association of the metabolite's concentration with each SNP in the region. Bottom: LD between each pair of SNPs in the region, with the colour scale for 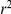 superimposed.

**Figure 3:**
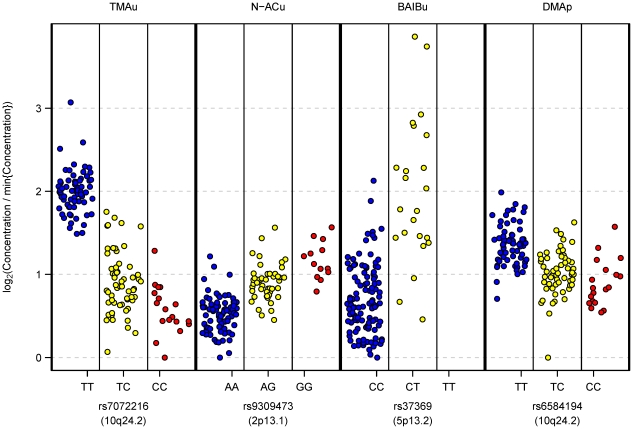
Relative metabolite concentrations against genotypes at their most significantly associated mQTL SNP. Each point corresponds to a study participant's mQTL genotype and corresponding metabolite concentration. Metabolite identifiers are labelled at top. Genotypic classes for each mQTL are shown on the horizontal axis (random horizontal variation within each genotypic class is introduced for clarity); dbSNP identifiers are labelled at bottom. At each metabolite peak, the transformed data vector shown in the plot is 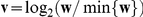, where 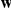
denotes the vector of normalized peak heights at that peak (prior to any logarithmic transformation, as described in *Materials and Methods*). So, the transformation maps to zero the lowest observed concentration of each metabolite, and log_2_(fold change) can be visually quantified relative to this baseline level. In particular, the maximum observed log_2_(fold change) in a metabolite's concentration is easily accessible from the plot. Within-participant replicate observations (biological and technical) were averaged on log_2_ scale.

Under this model, we calculated p-values for the test of no association between the metabolite and each regional SNP in turn. Figure 1, Figure 2, Figures S2 and S3 display the p-values for all regional tests of association superimposed on patterns of LD and the positions of genes. The details of association of each metabolite with its most strongly associated SNP are listed in Table 3, while Table S1 contains association results for SNPs within 200 kb of hit regions. The relationship between metabolite concentration and genotype is presented graphically in Figure 3.

**Table 3:** Statistical characterization of ^1^H NMR mQTL effects.

	Discovery Stage (MolTWIN)			Replication Stage (MolOBB)			
ID	Beta^a^	S.E. Beta	p-value	Beta	S.E. Beta	p-value	Replicated^b^
TMAu	-1.10	0.08	2.8E-23	-1.19	0.12	7.9E-15	*
N-ACu	1.06	0.09	4.1E-19	1.10	0.13	1.4E-11	*
BAIBu	1.54	0.21	5.9E-11	1.25	0.23	1.1E-06	*
DMAp	-0.65	0.09	8.6E-11	-0.52	0.19	0.0081	*

a Additive genetic effect with increasing number of copies of the minor allele (minor/major alleles are shown in Table 1).

b Significant at a level of 0.0125 (significance level of 0.05 adjusted for conducting 4 tests by the Bonferroni method).

### Variance decomposition of metabolite concentrations (^1^H NMR and Biocrates)

**Figure 4:**
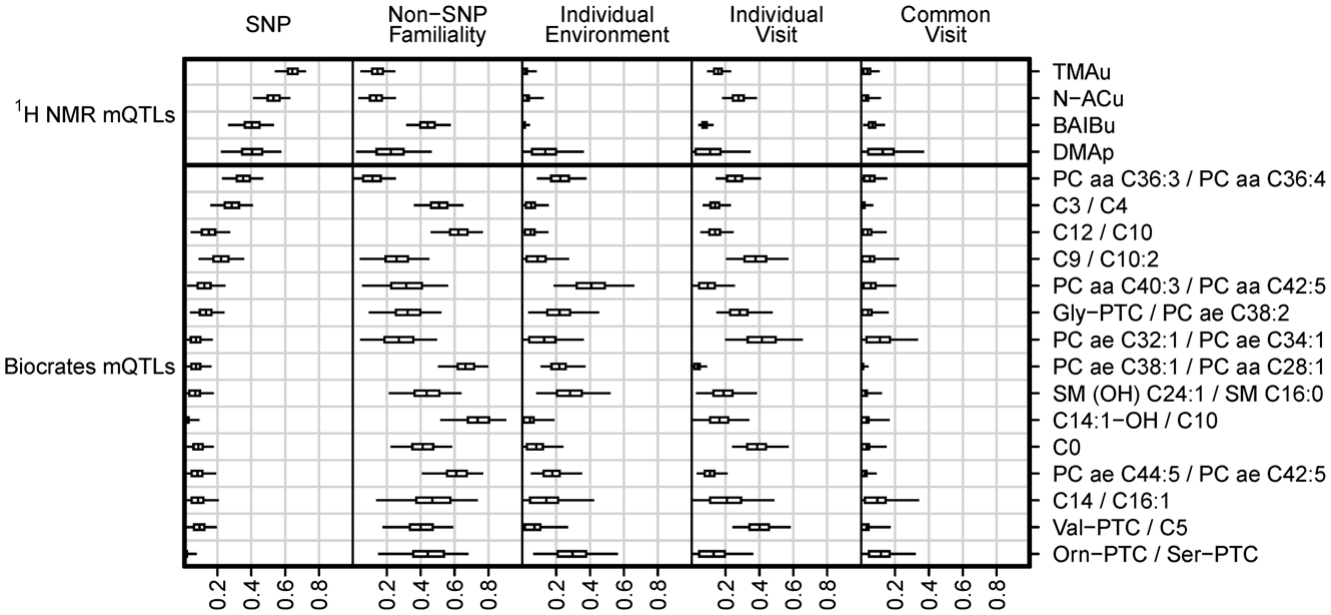
Biological variance decomposition for metabolic traits driven by mQTLs featuring in the current paper. Results from the current paper's replication of [14] on the Biocrates platform are shown in the bottom section of the plot. Results for ^1^H NMR mQTLs identified in the current study are shown in the upper section. For each metabolic trait (labelled right), the plot displays estimates of the proportion of biological variance explained by five complementary sources (labelled top; see *Materials and Methods* for explanation), including the mQTL SNP genotypes, familial variation excluding the mQTL SNP variation, individual environmental variation, and two types of visit variation (individual and common). Posterior distributions for proportions are represented as follows: the central tick in a box marks the posterior mean, the ends of a box mark the posterior quartiles, and the whiskers represent a 95% credible interval (extending to the 2.5 and 97.5 posterior percentiles).

For ^1^H NMR mQTLs, we estimated the proportion of biological variation in the metabolite's concentration explained by the corresponding mQTL SNP, and decomposed the remaining variation into familial, individual-environmental, and longitudinally fluctuating (visit) effects (Figure 4, Table 4, and *Materials and Methods*). The *familial* component of variation modelled the combined effects of genome-wide identity-by-descent genetic sharing, and common environment (i.e. environmental influences shared by twins after their conception). The individual-visit and common-visit components of variation modelled the longitudinal fluctuations between sample-donation visits that were respectively non-shared and shared by twins in a pair (the common-visit effect was included in the model because twins visited the clinic in pairs).

**Table 4:** Decomposition of biological population variation in metabolic traits.

				Percentage of Biological Variance Explained^a^				
Platform	Biofluid	Metabolic Trait^b^	SNP	mQTL SNP	Familiality^c^	Indiv. Envir.^d^	Indiv. Visit^e^	Common Visit
^1^H NMR	Urine	TMAu	rs7072216	64% (55–72)	15% (5–24)	2% (0–8)	16% (10–23)	4% (0–10)
^1^H NMR	Urine	N-ACu	rs9309473	53% (42–62)	14% (4–25)	3% (0–12)	28% (19–38)	3% (0–11)
^1^H NMR	Urine	BAIBu	rs37369	40% (27–53)	44% (32–57)	1% (0–4)	8% (5–12)	7% (2–13)
^1^H NMR	Plasma	DMAp	rs6584194	40% (23–57)	22% (3–46)	14% (0–36)	11% (0–34)	13% (0–36)
Biocrates	Plasma	PC aa C36:3/PC aa C36:4	rs174547	35% (23–46)	12% (1–25)	22% (9–37)	26% (15–40)	5% (0–15)
Biocrates	Plasma	C3/C4	rs2014355	29% (16–40)	51% (37–65)	5% (0–15)	14% (7–23)	1% (0–7)
Biocrates	Plasma	C12/C10	rs211718	15% (5–27)	62% (47–76)	5% (0–15)	14% (6–24)	4% (0–14)
Biocrates	Plasma	C9/C10:2	rs2286963	22% (10–35)	26% (5–45)	9% (0–27)	38% (21-56)	5% (0–22)
Biocrates	Plasma	PC aa C40:3/PC aa C42:5	rs9393903	12% (3–24)	32% (6–55)	41% (19–65)	10% (0–25)	6% (0–20)
Biocrates	Plasma	Gly–PTC/PC ae C38:2	rs2216405	13% (4–24)	32% (10–52)	22% (4–45)	28% (15–47)	4% (0–16)
Biocrates	Plasma	PC ae C32:1/PC ae C34:1	rs7156144	7% (1–17)	27% (5–49)	13% (0–36)	42% (20–65)	11% (0–33)
Biocrates	Plasma	PC ae C38:1/PC aa C28:1	rs11158519	7% (1–16)	66% (51–79)	22% (11–37)	3% (0–8)	1% (0–4)
Biocrates	Plasma	SM (OH) C24:1/SM C16:0	rs168622	7% (0–17)	44% (22–64)	28% (9–51)	19% (3–38)	2% (0–12)
Biocrates	Plasma	C14:1-OH/C10	rs8396	2% (0–9)	74% (52–90)	5% (0–18)	16% (1–33)	3% (0–16)
Biocrates	Plasma	C0	rs7094971	9% (2–17)	41% (23–58)	8% (0–24)	39% (25–57)	3% (0–14)
Biocrates	Plasma	PC ae C44:5/PC ae C42:5	rs2046813	8% (1–19)	61% (41–76)	18% (6–35)	11% (4–20)	2% (0–8)
Biocrates	Plasma	C14/C16:1	rs603424	9% (1–20)	47% (14–73)	14% (0–42)	21% (0–48)	10% (0–34)
Biocrates	Plasma	Val-PTC/C5	rs272889	9% (2–19)	40% (18–58)	7% (0–26)	40% (25–58)	3% (0–17)
Biocrates	Plasma	Orn-PTC/Ser-PTC	rs541503	2% (0–7)	44% (15–68)	30% (7–56)	13% (0–36)	12% (0–32)

a See *Materials and Methods* for the definition of each component of variance. Posterior mean estimates are shown with parenthesized central 95% posterior credible intervals.

b Supplementary material of [14] has details of the metabolites targeted by the Biocrates platform, and their abbreviations.

c Variation attributable to familial (i.e. heritable and common-environmental) sources, but not to the mQTL SNP itself.

d Individual environment.

e Individual visit.

The proportions shown in Figure 4 and Table 4 are proportions of phenotypic variance after the experimental variance has been removed. It was useful to extract the experimental variance prior to comparison across platforms, as the primary focus was on the variability properties of the metabolite concentrations, not on the experimental variation associated with the measurement process. The mQTLs explained 40%–64% of biological population variation in the corresponding ^1^H NMR metabolite levels.

We also performed a variance decomposition of the metabolic traits, quantified on the Biocrates platform, for which mQTLs were identified in [14] (Figure 4, Table 4, and *Materials and Methods*). The Biocrates-platform mQTLs explained up to 35% of biological variation in the corresponding metabolic traits (smaller effect sizes than for the ^1^H NMR mQTLs). Our results qualitatively extended the findings of [14]: the current study's design allowed the decomposition of the component of variation in metabolite concentration that was *not* explained by the mQTL itself (see Discussion).

To investigate potential bias in effect-size estimates (the “winner's curse” phenomenon [27]), we compared effect-size estimates across discovery and replication studies, both for the Biocrates-platform mQTLs (Figure S4), and for the ^1^H NMR mQTLs (Figure S5). We found there to be a good degree of consistency in effect-size estimates between discovery and replication studies.

### Quantification of study power (^1^H NMR and Biocrates)

**Figure 5:**
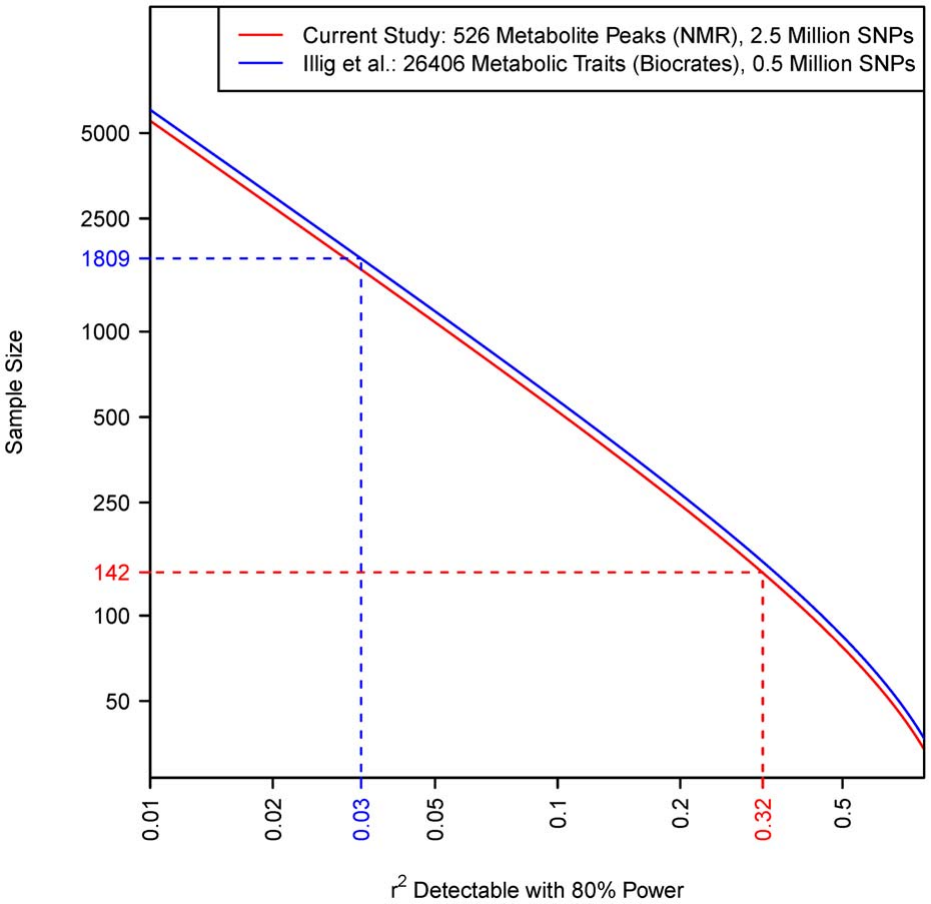
Relationship between sample size and the size of effect detectable with 80% power in each study (shown by solid lines). The effect size is parameterized by 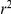, which is the proportion of total population variance in metabolite concentration explained by the mQTL genotype (or, equivalently, the squared correlation between genotype and trait). It is assumed that the family-wise error rate in each study is controlled at 0.05 using the Bonferroni method. The number of tests performed is calculated as the product of the SNP and metabolite counts, as shown in the legend. Dashed lines relate the actual sample size of each study to that study's detectable effect size.

Figure 5 relates the detectable effect size (the proportion of variance in concentration explained by the mQTL SNP, quantified by 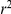) to the sample size for each study (power calculations used the GeneticsDesign R package). Our study had power to detect associations with approximately 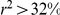, while [14] had power to discover much smaller effects (approximately 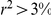). Better powered studies such as [14] have the potential to offer further interesting insights into the mQTL basis of the ^1^H NMR metabolome.

### Proximity of mQTLs to known GWAS SNPs (^1^H NMR)

We searched within 200 kb of each metabolite's hit region for SNPs previously associated with phenotypes in GWASs [28]. SNP rs13538 is in strong LD with the N-ACu hit region at chromosome 2p13.1 (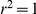 between rs13538 and rs9309473 in the HapMap 3 individuals of Northern European ancestry, i.e. HapMap-CEU [29]). Variation at rs13538 has been shown to correlate with serum creatinine concentration and other measures of renal impairment, as well as with susceptibility to chronic kidney disease [22]–[23].

### Coincidence of mQTLs with positively selected regions (^1^H NMR and Biocrates)

Upon surveying the literature related to genes in the region of the N-ACu mQTL, we realized that several papers had highlighted this particular region as carrying one of the strongest signatures of selection that has been discovered in the human genome (see, e.g., [8], [30]). This led us to check all known mQTLs for coincidence with positively selected regions (as identified by the genome-wide scan for such regions in [8]; see also [31] for a review of the detection and relevance of the genetic signature of natural selection).

We compared the locations of all mQTLs discussed in the current paper to the positively selected loci identified in [8]. Two of our three replicated mQTL hits were within such regions (the mQTL for N-ACu, and the mQTL that affects both TMAu and DMAp). We also examined the genomic locations of each of Illig et al.'s 13 replicated mQTLs (see dedicated section below on replication), and found none to be within positively selected regions as identified in [8].

### Analysis of TMAu and DMAp mQTL (^1^H NMR)

SNPs significantly associated with TMAu and DMAp fall within a haplotype block of approximately 40 kb at chromosome 10q24.2, which contains the *PYROXD2* gene, a probable pyridine nucleotide-disulphide oxidoreductase gene, previously named *C10orf33* (see Figure 2 and Figure S2). The most strongly associated SNP, rs7072216, has alleles C/T at frequency 0.25/0.75 in Europe (HapMap-CEU [29]). Our data indicate that TMAu concentration and DMAp concentration both increase with the number of copies of the major (T) allele. TMAu displays non-additivity, with the T allele recessive, and the TT homozygote class showing a greater-than-additive increase (on logarithmic scale) on the levels of the other two genotypic classes (Figure 3). There is a non-synonymous SNP—rs2147896—in strong LD with rs7072216 (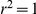; see also Table S2A). Functional predictions (SIFT [32] and PolyPhen [33]) and the PhyloP conservation score [34]–[35] for rs2147896 did not point to a clear functional impact, or to it being significantly conserved (Table S2B). SNP rs2147896 does not lie in a known protein domain, and web-based protein structure-modelling tools [36]–[37] did not predict that the rs2147896 polymorphism would have an effect on PYROXD2's ligand binding site. However, *PYROXD2* (*C10orf33*) eQTLs have been discovered in fibroblasts (rs2147897 [2]) and liver (rs2147901 [38]), with these eQTLs in high LD (up to 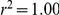 and 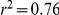 respectively) with mQTL SNPs of TMAu and DMAp (Table S3). This raised the possibility that eQTL-driven population variation in mRNA transcription at *PYROXD2* mediates the mQTL of TMAu and DMAp.

**Figure 6:**
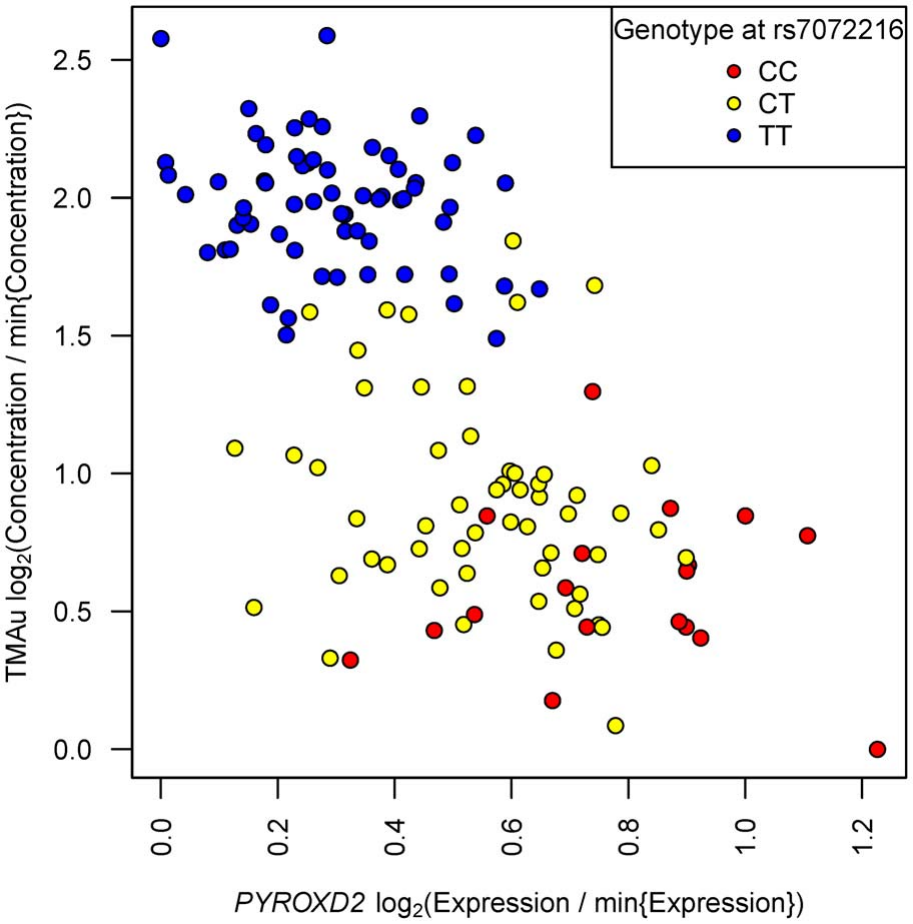
TMAu's mQTL effect may be mediated by variation in mRNA transcription at *PYROXD2*. Each point represents, for a single study participant, their concentration of TMAu (vertical axis), their expression of *PYROXD2* in adipose tissue (horizontal axis), and their mQTL genotype (point colour). The intensity data, 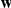, on each of the vertical and horizontal axes have been transformed 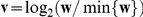. This transformation sets the minimum observation to zero on log_2_ scale, and presents log_2_(fold change) relative to the minimum value.

In order to investigate this eQTL hypothesis further, we extracted estimates of *PYROXD2* mRNA abundance from two separate gene-expression microarray data sets measured on abdominal subcutaneous adipose tissue and whole-blood samples from the MolTWIN cohort (*Materials and Methods*). We found that *PYROXD2* was expressed in whole blood, but found no evidence of rs7072216 being an eQTL of *PYROXD2* in whole blood (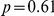), a finding consistent with [2], in which *PYROXD2* eQTLs were neither discovered in T cells nor in lymphoblastoid cell lines. However, we did find rs7072216 to be an eQTL of *PYROXD2* in subcutaneous abdominal adipose tissue (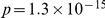), with gene expression decreasing in the number of copies of the T allele. We plotted the mutual dependence between rs7072216 genotype, *PYROXD2* gene expression in adipose tissue, and TMAu concentration (Figure 6). TMAu concentration was strongly negatively correlated with *PYROXD2* expression (Pearson's 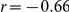, 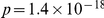).

We examined these particular gene expression data (i.e. measured in fat and blood cells) because they had been acquired already on MolTWIN cohort members. In performing this analysis, we were not suggesting that variation in gene expression in fat has a direct impact on the concentration of TMAu or DMAp. However, a substantive proportion of eQTLs modulate expression in a similar way in different tissues [39]. Thus, in identifying and characterizing the mutual dependence of TMAu concentration, rs7072216 genotype, and *PYROXD2* expression in a mechanistically unrelated tissue (i.e. fat), we have raised the possibility that a qualitatively similar relationship with *PYROXD2* expression will be observed in the tissue that truly mediates the mQTL effect (likely to be liver or kidney).

### Analysis of BAIBu mQTL (^1^H NMR)

The SNPs that are significantly associated with BAIBu map to chromosome 5p13.2 within *AGXT2* (alanine-glyoxylate aminotransferase 2). AGXT2 is known to be expressed in human liver and kidney. An eQTL for *AGXT2* was reported in liver ([38] and Table S3), but this eQTL is not in LD with the mQTL SNPs (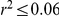), and so does not explain the BAIBu mQTL.

Two of the most significant mQTL SNPs for BAIBu were rs37369 (T/C at 0.09/0.91) and rs37370 (C/T at 0.08/0.92), with 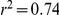 (HapMap-CEU [29]) between the two SNPs (Table S2A). At SNP rs37370, one of the MZ twin pairs in the study was homozygous for the minor C allele; these subjects had higher BAIBu concentration than those in the other genotypic classes. Each of rs37369 and rs37370 is a non-synonymous, missense coding mutation in *AGXT2*, leading to an amino acid substitution in AGXT2. At rs37369, the base change C619T leads to the valine-to-isoleucine substitution V140I. At rs37370, T506C leads to the asparagine-to-serine substitution N102S. At each SNP, the concentration of BAIBu increased in the number of copies of the minor allele. Both SNPs lie in the pyridoxal phosphate-dependent transferase major domain (IPR015424) with rs37369 in subdomain 1, and rs37370 in subdomain 2. We extracted functional predictions (SIFT [32] and PolyPhen [33]) and PhyloP conservation scores [34]–[35] for rs37369 and rs37370, but discovered no substantive evidence in favour of functional impact or of either SNP being significantly conserved (Table S2B). We used the web servers Phyre2 [36] and 3DLigandSite [37] to predict AGXT2 protein structure and to investigate whether rs37369 and rs37370 were likely to affect AGXT2's predicted ligand binding site, but neither SNP was identified in these analyses as having an impact on the binding site.

### Replication of Illig et al. (Biocrates)

We analysed the 15 mQTL associations reported in Illig et al. [14] using SNP genotypes and Biocrates-platform data from the MolOBB and MolTWIN cohorts (having removed individuals overlapping with the TwinsUK cohort used in [14]). We replicated 12 of the 15 mQTLs (Table 5), with four additional mQTLs replicated beyond the nine replicated by Illig et al. themselves (so that now a total of 13 of the 15 mQTLs identified in [14] have been replicated). The same significance level was used as in the replication section of [14], specifically a level of 0.05 adjusted by the Bonferroni method to account for 15 tests being performed (i.e. an adjusted significance level of 0.0033).

**Table 5:** Summary of the current study's replication of Illig et al.'s [14] mQTL associations (Biocrates platform).

							Illig et al.^a^		Current Paper		Replicated^b^	
SNP	Gene	Chr	Position^c^	Ref./Alt.^d^	MAF^e^	Metabolic Trait^f^	Beta^g^	p-value	Beta	p-value	Illig et al.^h^	Current Paper
rs174547	FADS1	11	61327359	T/C	0.304	PC aa C36:3/PC aa C36:4	0.151	6.5E-179	0.161	1.3E-20	*	*
rs2014355	ACADS	12	119659907	T/C	0.277	C3/C4	−0.218	5.1E-96	−0.254	5.7E-13	*	*
rs211718	ACADM	1	75879263	C/T	0.305	C12/C10	0.12	1.3E-63	0.098	3.7E-06	*	*
rs2286963	ACADL	2	210768295	T/G	0.365	C9/C10:2	0.219	3.1E-60	0.169	4.1E-06	*	*
rs9393903	ELOVL2	6	11150895	G/A	0.246	PC aa C40:3/PC aa C42:5	0.087	2.3E-42	0.072	9.9E-05	*	*
rs2216405	CPS1	2	211325139	A/G	0.185	Gly-PTC/PC ae C38:2	0.129	1.9E-30	0.224	2.0E-06		*
rs7156144	PLEKHH1	14	67049466	G/A	0.414	PC ae C32:1/PC ae C34:1	−0.042	1.7E-28	-0.037	5.0E-04	*	*
rs11158519	SYNE2	14	63434338	G/A	0.145	PC ae C38:1/PC aa C28:1	−0.083	1.5E-27	-0.094	7.7E-05		*
rs168622	SPTLC3	20	12914089	G/T	0.375	SM (OH) C24:1/SM C16:0	0.061	5.2E-26	0.045	2.0E-03	*	*
rs8396	ETFDH	4	159850267	T/C	0.298	C14:1-OH/C10	0.102	3.5E-24	0.066	3.9E-02	*	
rs7094971	SLC16A9	10	61119570	A/G	0.135	C0	−0.091	3.8E-20	−0.104	3.0E-05	*	*
rs2046813	ACSL1	4	186006153	T/C	0.322	PC ae C44:5/PC ae C42:5	0.033	3.6E-18	0.042	2.7E-03		*
rs603424	SCD	10	102065469	G/A	0.194	C14/C16:1	0.054	1.5E-17	0.053	1.1E-02		
rs272889	SLC22A4	5	131693277	G/A	0.385	Val-PTC/C5	−0.075	7.9E-16	-0.102	2.5E-04		*
rs541503	PHGDH	1	120009820	T/C	0.379	Orn-PTC/Ser-PTC	0.058	3.0E-12	0.039	2.2E-01		

a Estimate and p-value from the discovery stage of [14] (their KORA cohort).

b Significant at a level of 0.0033 (significance level of 0.05 adjusted for conducting 15 tests by the Bonferroni method).

c NCBI build 37 coordinates.

d Reference/alternative Allele.

e Minor allele frequency, from Table 1 of [14].

f Supplementary material of [14] has details of the metabolites targeted by the Biocrates platform, and their abbreviations.

g Beta is the estimate of the additive effect of one copy of the reference allele, normalized by the mean of the metabolic trait.

h Significant in the replication stage of [14] (their TwinsUK cohort); see Table 1 of [14] for details.

## Discussion

The current paper has extended recent studies [14], [19] in investigating the genetic basis of human metabolism. We analysed plasma and urine samples using ^1^H NMR, whilst Illig et al. [14] analysed serum samples using the Biocrates platform (targeted metabolomics using FIA-MS) [18]. While our examination of urine metabolites did not overlap with previous work, there was some minimal overlap between the metabolites targeted in blood (plasma or serum) by Biocrates and ^1^H NMR [40]. The Biocrates platform focuses specifically on a pre-selected set of amino acids and lipids [14], [18]. In contrast, ^1^H NMR spectroscopy is untargeted, quantifying the most abundant 50–100 metabolites in a biofluid, typically those above 10 micromolar in concentration [15]. We were able to annotate 38 metabolites in our plasma ^1^H NMR data, of which five were also targeted by the Biocrates platform (glutamine, glycine, leucine, tyrosine and valine). So, the sets of metabolites considered by the two studies are minimally overlapping and therefore complementary.

The MolTWIN plasma and urine samples were collected longitudinally from twins, and analysed with technical replication using ^1^H NMR and the Biocrates platform. This study design permitted a detailed decomposition of population variance in metabolite concentration (Figure 4 and Table 4). We estimated the proportion of biological variation in metabolite concentration explained by the corresponding mQTL SNP (*biological* variation included all phenotypic variation apart from that which was experimentally derived). For the newly discovered ^1^H NMR mQTLs, this proportion varied between 40%–64%. For the 13 currently replicated mQTLs discovered by Illig et al. [14], the proportion varied between 2%–35%. This discrepancy is explained by the different study designs, and mainly by the different sample sizes (Figure 5).

The current study's twin design allowed us to quantify the proportion of biological variance in metabolite concentration that was attributable to familial factors (i.e. genetic and common-environmental effects). For Illig et al.'s replicated mQTLs, the ‘non-SNP’ familial variation (i.e. familial variation not explained by the mQTL) was considerable, explaining on average 44% (range 9%–70%) of biological variation in the corresponding metabolic trait (Figure 4 and Table 4). On average, Illig et al.'s mQTL SNPs explained 25% (range 5%–82%) of the total familial variation in the corresponding metabolic traits. So, other genetic and common-environmental factors had substantial influence in addition to (and perhaps interacting with) the effects of the mQTLs themselves.

In a separate study [41], we have characterized population variation in all common ^1^H NMR-detectable urine and plasma metabolites, using the MolTWIN ^1^H NMR data of the current study, though without incorporation of the genotype data. We decomposed biological population variation into components, including that explained by familial sources and that explained by longitudinally stable sources. On average (across ^1^H NMR peaks), familial sources explained 42% (IQR 32-52) of variation in plasma metabolite concentrations and 30% (IQR 17-39) of variation in urine ones. Longitudinally stable sources explained 60% (IQR 51-72) of variation in plasma metabolite concentrations and 47% (IQR 35-60) of variation in urine ones. The substantive widespread presence of familial and stable variation across the urine and plasma ^1^H NMR metabolomes has implications for the design and interpretation of metabolite biomarker-discovery studies [41].

Interestingly, two of the ^1^H NMR mQTLs (discussed in detail below) have experienced recent positive selection in European populations [8]. The fact that these mQTL SNPs experienced selection suggests that molecular and phenotypic perturbations downstream of them may be biomedically interesting [31]. Also, the identification of functional consequences of variation at these loci strengthens the existing genetic evidence for selection having acted at these loci [31]. The observed genetic signature of positive selection suggests that an allele at the locus conferred a net advantage, relative to other alleles, under some environmental pressures, yet did not confer a net advantage under other environmental pressures. Humans may still be exposed to relevant environmental heterogeneity, and so the biomedical implications of these loci may become most clear once gene-environment interactions are incorporated into disease-susceptibility models. It will be initially of interest to investigate how physiological metabolite concentrations vary between world-wide populations as a result of different mQTL allele frequencies and environmental backgrounds. Metabolic profiles have the potential to reflect the synergy of genetic and environmental influences, and can thus provide unique insights into disease susceptibility at a population level [42]–[43].

During the revision stage of the current paper, an article by Suhre et al. [44] appeared, describing a GWAS of urine metabolite concentrations targeted by the ^1^H NMR-based Chenomx platform. Only one of the three mQTLs identified in the current paper—that of BAIBu—was identified in [44] (see further discussion of the BAIBu mQTL in the dedicated section below). A comparison of the current study with [44] illustrates nicely some of the differences between targeted and untargeted assays. In the current study we searched for strong genetic drivers of the comprehensive set of common urine and plasma metabolites detectable by ^1^H NMR. In contrast, Suhre et al. investigated genetic drivers of a targeted subset of the urine ^1^H NMR metabolome with a substantially greater sample size than that of the current study, and thus had statistical power to detect relatively weak genetic effects. The untargeted nature of the current study allowed the detection of two strong mQTL drivers of urine metabolite concentrations—TMAu and N-ACu—that were not targeted by the Chenomx platform used in [44]. A disadvantage of our untargeted approach in this context was that peaks had to be annotated with their corresponding metabolite. In the current study we were unable to attribute N-ACu to a single metabolite, and so the N-ACu mQTL was reported as driving concentrations of one or more *N*-acetylated compounds (X.NH.CO.CH_3_, with X unknown). Also, the unambiguous annotation of BAIBu was assisted by input from Suhre et al. [44], previous to which we had annotated the peak non-uniquely as CH_3_.CH.Y, with Y unknown but containing CH or CH_2_ and an electronegative substituent.

### Discussion of TMAu and DMAp mQTL (^1^H NMR)

Genetic variation at *PYROXD2* has experienced recent positive selection in European populations [8], with the T allele of rs7072216 at frequency 0.75 in Europe (HapMap-CEU), and at 0.14 in Africa (HapMap 3 individuals from Yoruba in Ibadan, Nigeria, Africa, i.e. HapMap-YRI [29]). The haplotype that was relatively advantageous in European populations is associated with decreased expression of *PYROXD2* and increased concentration of TMAu and DMAp. Further work will be necessary to clarify the mechanisms linking: DMAp and TMAu levels; *PYROXD2* gene expression; and genetic variation in LD with rs7072216 (such as the non-synonymous SNP, rs2147896). The signature of selection at *PYROXD2* is indirectly suggestive of biomedical relevance; we also note that the set of genes showing evidence for positive selection is enriched for genes involved in oxidoreductase activity [8].

There have been a number of studies that have examined the sources of variation in physiological concentrations of methylamines and their derivatives, e.g. [45]–[46]. The current paper sheds light on this field from a new genetic angle, and it will be useful to integrate the mQTL effects into known pathways. Gut microbiota play an important role in the formation of methylamines from dietary sources in mammals—they create TMA from choline, and convert TMA into DMA [45]–[46]. Trimethylamine *N*-oxide (TMAO) is formed endogenously in the liver via the *N*-oxygenation of TMA by the flavin-containing monooxygenase (FMO) protein family, and particularly by FMO3 [47]. Gut microbial activity has been linked to disease through physiological levels of DMA, TMA and TMAO [48]–[49].

It may prove productive to relate the TMAu mQTL finding to the rare recessive genetic disorder trimethylaminuria, in which mutations at *FMO3* disrupt conversion of TMA to TMAO, resulting in high physiological levels of TMA and an accompanying fish-odour phenotype [47]. Trimethylaminuria cases exhibit relatively low values of the ratio TMAOu/(TMAOu + TMAu), where TMAOu denotes urine TMAO concentration. Subjects in the current study have values of this ratio that are within the range typical of trimethylaminuria controls (Figure S6 and [50]). It will be interesting to investigate the effect, if any, of genetic variation at the TMAu mQTL on TMA levels among trimethylaminuria cases.

### Discussion of N-ACu mQTL (^1^H NMR)

The N-ACu mQTL lies within a large 500 kb haplotype block (Figure 1), and there are a number of genes (and eQTLs, Table S3) in LD with it. Of these genes, *NAT8* is a likely candidate for mediating the association between SNP variation and N-ACu (urine concentration of *N*-acetylated compound(s)), since *NAT8*'s encoded enzyme specifically catalyzes *N*-acetylation—*NAT8*'s enzyme is cysteinyl-conjugate *N*-acetyltransferase, CCNAT [51]. We relate our N-ACu mQTL finding to other research that has shown: (i) that the region harbours SNPs associated with renal function [22]–[23], [52]; and (ii) that the region has been the site of positive selection on standing genetic variation [8], [30].

Two recent renal-function GWASs identified rs13538 as a clinically associated SNP [22]–[23], [52] with the minor G allele increasing susceptibility to renal dysfunction. Chambers et al. [22] proposed that a non-synonymous mutation in *NAT8* (the A595G change at rs13538, producing a non-conservative amino acid change F143S in CCNAT) *reduces* acetylation efficiency, thus leading to toxin-induced kidney injury. The N-ACu mQTL SNP rs9309473 is in strong LD (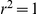) with GWAS SNP rs13538 (both SNPs with alleles A/G at frequency 0.79/0.21 in HapMap-CEU [29]). We found the non-synonymous mutant allele (G) at rs13538 to be associated with *increased* levels of N-ACu. Thus, whilst our findings provide evidence of differential acetylation efficiency driven by genetic variation in LD with rs13538, their directionality is not consistent with the specific mode of action proposed in [22]. Furthermore, a recent functional study [51] found enzymatic activity of mutant (F143S) CCNAT to be comparable to that of the wild-type protein (and so is also inconsistent with the mode of action proposed in [22]).

Scheinfeldt et al. [30] studied the signature of selection in this region, specifically examining two complementary sets of haplotypes: the “ancestral” and “derived” haplogroups (HapA/HapD respectively, at frequency 0.26/0.74 in the HapMap-CEU European population, but at 0.89/0.11 in the HapMap-YRI African population [29]). It has been proposed that positive selection drove up the frequency of HapD (relative to HapA) in Eurasian populations about 15,000 years ago [30]. The N-ACu mQTL SNP rs9309473 is in strong LD with HapA/HapD status (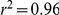 in HapMap-CEU, with alleles G/A of rs9309473 highly predictive of HapA/HapD status respectively). An increasing number of copies of HapA is associated with increased urine concentration of *N*-acetylated compound(s) (N-ACu), and with increased susceptibility to renal dysfunction [22]–[23].

### Discussion of BAIBu mQTL (^1^H NMR)

The BAIBu mQTL was also identified by Suhre et al. [44], where they noted the following. Elevated levels of BAIBu had been shown through family studies to be autosomal recessive [53], but the causal locus had been previously unknown. The association of a SNP in *AGXT2* with BAIBu levels is consistent with the role of *AGXT2*'s encoded enzyme, mitochondrial aminotransferase, which is expressed primarily in the kidney and catalyzes the reaction of BAIB with pyruvate to form 2-methyl-3-oxopropanoate and alanine (EC 2.6.1.40). It had also been previously suggested that altered BAIB homeostasis might contribute to hyper-β-amino-isobutyric aciduria, a relatively common Mendelian metabolic disorder in humans [54]. Suhre et al. [44] proposed rs37369 as a likely candidate for the causative SNP driving both variation in BAIBu concentration and susceptibility to hyper-β-amino-isobutyric aciduria.

We found the non-synonymous SNP rs37369 (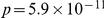) to be marginally more significantly associated with BAIBu concentration than the other non-synonymous SNP, rs37370 (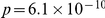). This mildly supports rs37369 as the causal SNP driving BAIBu levels, relative to rs37370, though the true causal genetic polymorphism may be neither of these SNPs, but instead variation in strong LD with them. We used existing tools to predict the effect of rs37369 and rs37370 polymorphism on AGXT2 function (see Results), but this analysis did not reveal any clear functional consequences of these non-synonymous polymorphisms. Further work will be necessary to characterize with certainty the causal link between genetic variation at the *AGXT2* locus and BAIBu concentration.

### Conclusion

In conclusion, we have designed and conducted an mQTL study of plasma and urine metabolites detectable by ^1^H NMR. We discovered and replicated four novel metabolite-SNP associations, with each SNP explaining 40% or more of biological variation in metabolite concentrations. The mQTLs that we discovered have interesting properties: two of the three mQTL regions have experienced recent positive selection in European populations; one mQTL is in strong LD with a SNP identified in a kidney-function GWAS. Our findings pave the way forward for investigating the potential biomedical relevance of these regions.

## Materials and Methods

### Ethics statement

The MolTWIN study was approved by St. Thomas' Hospital Research Ethics Committee (EC04/015 Twins UK). The MolOBB study received ethical approval from Oxfordshire REC C (08/H0606/107).

### Participant recruitment—MolTWIN

The 142 participants in the current study were recruited from the UK Adult Twin registry at St. Thomas' Hospital (www.twinsUK.ac.uk): a longitudinal epidemiological study of 11,000 twins (mostly female), for which extensive clinical, anthropometric, lifestyle, and demographic information, and a wide range of biological measurements have been collected [55]. Eligible volunteers were healthy, Caucasian, post-menopausal females of Northern European descent, between 45–76 years of age. Eligible twins were sent an information sheet containing details of the study, and two consent forms. After each twin had returned a completed consent form, she was contacted by letter and phone to book her appointment. The composition of the cohort was: 51 MZ pairs, 19 DZ pairs, and two unrelated individuals.

In the MolTWIN cohort, 33 of the MZ twin pairs donated samples twice; the median inter-visit time across all such pairs was 118 days (IQR: 96-134). Both twins in a pair always visited on the same day, and each visit was scheduled at either 10:00 or 14:00 (with repeated visits of each individual not necessarily scheduled at the same time of day).

### Participant recruitment—MolOBB

The 69 participants in the current study were selected from the Oxford Biobank [20] (OBB). Specific OBB cohort members were selected on the basis of case/control status for metabolic syndrome according to International Diabetes Foundation Criteria [56]. The set of subjects comprised 42 controls (17 female, 25 male), and 27 cases (12 female, 15 male).

### Sample collection

Fasting blood and urine samples were collected at all clinic visits of each participant. Spot urine samples were centrifuged (16060 × g) at 4°C for 10 min before being stored at −80°C. Fresh blood was collected in a 9 mL tube through venepuncture. Samples for ^1^H NMR analysis were collected in heparin tubes, whilst samples for Biocrates-platform analysis were collected in EDTA tubes. Blood samples were kept on ice for 20 min prior to centrifugation (16060 × g) at 4°C for 10 min, and subsequent storage at −80°C.

### Genotyping, quality control, and imputation (MolTWIN and MolOBB)

DNA was extracted from whole-blood samples using GeneCatcher (Invitrogen Life Technologies, Carlsbad, USA) according to manufacturer's protocol. Genome-wide SNP genotypes were measured on a total of 166 individuals: 70 from the MolOBB cohort, and 96 from the MolTWIN cohort (one MZ twin from each MZ pair was genotyped, whilst both members of each DZ twin pair were genotyped). The genotyping assay used was the Illumina 317K BeadChip SNP array (Illumina, San Diego, USA).

Quality control on the genotyped subjects was performed in a way similar to those described previously by the Wellcome Trust Case Control Consortium [57]. Two MolTWIN samples were removed due to sample genotyping success rate < 95% and three samples (two from MolTWIN, one from MolOBB) were removed due to non-European ancestry (note that the cohort compositions given in the Participant Recruitment sections are after quality control). SNPs were removed (i) if MAF < 1%, or (ii) if genotyping success rate <95% and MAF > 5%, or (iii) if genotyping success rate <99% and MAF < 5%. Hardy-Weinberg equilibrium (HWE) was calculated by combining all unrelateds of the MolOBB and MolTWIN data sets (i.e. one twin per twin pair) and the hypothesis of HWE was tested at a significance level of 10^−4^; SNPs at which HWE was rejected were omitted from the study. After quality control, the genotypes of ungenotyped MZ twins were copied from their corresponding genotyped twin. The final data set prior to imputation comprised 69 MolOBB members and 142 MolTWIN members genotyped at 296,017 autosomal SNPs.

Measured genotypes were used to impute an additional 2,245,627 SNPs using the HapMap-CEU population (release 22) as reference [29]. The imputations were performed using IMPUTE [58]. We included SNPs in our analysis only if the imputation quality score was greater than 0.4. As output for a single SNP in an individual, IMPUTE provided probabilities of the individual having each of three possible genotypes (zero, one, or two copies of the reference allele). Prior to incorporating imputed genotypes into the statistical models, we preprocessed them, estimating the true genotype by that which was allocated highest probability by IMPUTE. Including both typed and imputed SNPs, we used a total of 2,541,644 autosomal SNPs for association analysis.

### Sample preparation, data acquisition, and preprocessing (MolTWIN gene-expression data)

Total RNA was extracted from adipose tissue biopsies with TRIreagent (SIGMA-ALDRICH, Gillingham, UK) and quantified using a NanoDrop. For whole-blood samples, PAXgene tubes were used, and RNA was extracted according to the manufacturer's protocol (PAXgene, QIAGEN). RNA was labelled using the MessageAmp II 96-well amplification kit (Applied Biosystems, CA, USA). Labelled RNA was hybridized onto Affymetrix HGU133 Plus2 arrays, washed, stained, and scanned for fluorescence intensity according to manufacturers protocols (Affymetrix, Inc., USA).

Data were preprocessed using the RMA method without background correction (i.e. quantile normalization followed by robust probe-set summarization) [59]. Whole-blood array data were preprocessed separately from adipose-tissue array data. Publicly available custom chip-definition files (CDFs) were downloaded (version 11) (http://brainarray.mbni.med.umich.edu/Brainarray/Database/CustomCDF/CDF_download.asp) and used to group probes into sets, each set corresponding to an Ensembl-annotated gene, resulting in 18,394 such genes represented in the array data. See [60] for a description of how these CDFs were created, along with a comparison of their properties with the CDFs produced by Affymetrix.

Expression data were extracted at the *PYROXD2* gene, and used in the current paper's analysis of the mQTL for TMAu and DMAp.

### Sample preparation and data acquisition (Biocrates)

EDTA plasma samples were vortexed after thawing and centrifuged at 4°C for 5 min at 10,000 x g prior to loading of 10 µL of supernatants onto the 96-well kit plate. Processing of the AbsoluteIDQ kit followed the protocol specified by the manufacturer, including the following automated steps on the Hamilton ML Star robotics platform (Hamilton Bonaduz AG, Bonaduz, Switzerland): (i) drying plasma samples under a nitrogen stream, (ii) derivatization of amino acids with 5% phenylisothiocyanate reagent (20 µL), (iii) drying of samples, (iv) extraction of metabolites and kit internal standards with mM ammonium acetate in methanol (300 µL), (v) centrifugation through filter plate (2 min, 500 x g), vi) dilution with 600 µL MS running solvent. 20 µL of the final extracts were applied to flow injection analysis mass spectrometry.

Samples were analyzed using an API 4000 triple quadrupole mass spectrometer (ABSciex) equipped with an Agilent 1200 Series HPLC and a HTC PAL auto sampler from CTC controlled by the software Analyst 1.5. The standard flow injection method comprising two 20 µL injections (one for positive and one for negative electrospray ionisation mode) was applied for all measurements. Quantification was achieved by multiple reaction monitoring detection in combination with the use of stable isotope-labelled and other internal standards [61]. Data evaluation for quantification of metabolite concentrations was performed with the MetIQ software package (integral part of the AbsoluteIDQ kit). Concentrations of all metabolites are initially calculated in µM. The method has been proven to conform to FDA-Guidelines [62], which imply proof of reproducibility within a given error range. Analytical specifications for detection limit (LOD) and evaluated quantification ranges, further LOD for semi-quantitative measurements, identities of quantitative and semi-quantitative metabolites, specificity, potential interferences, linearity, precision and accuracy, reproducibility and stability were described in Biocrates manual AS-P150. The LODs were set to three times the values of zero samples. The lower and upper limits of quantification were determined experimentally by Biocrates AG (Innsbruck, Austria). In addition, the technical variability of the Biocrates platform had been quantified previously by Illig et al. [14]. Their Supplementary Table 4 displayed the coefficient of variation, CV, for each of 163 metabolite concentrations assayed in [14], and measured under the same conditions on the same platform in the current study. The median CV across metabolites was 7.4% (IQR: 6.1%-12.4%) [14], which demonstrated a useful degree of precision for the majority of metabolites.

We performed quality-control checks, including boxplots and principal-component score plots, on the Biocrates-platform data to identify failed assays, where an *assay* refers to the measurement of 163 metabolite concentrations in a biological sample. Of a total of 356 assays across the MolOBB and MolTWIN cohorts, we identified two assays that exhibited anomalously low concentrations of all metabolites (relative to the levels observed in the other assays); we omitted those two assays from further analysis.

### Sample preparation and data acquisition (^1^H NMR)

Thawed samples were centrifuged at 16060 × g for 10 min. Samples were aliquotted into two technical replicates prior to sample preparation. Plasma was diluted 1 in 4 in physiological saline prepared in 20% D_2_O supplemented with 0.1% (w/v) sodium azide as a bacteriostatic agent and 1.5 mM sodium formate as a chemical-shift reference (δ8.452). Urine was diluted 2 in 1 in phosphate buffer (20% D_2_O, pH 7.4) supplemented with 1 mM trimethylsilyl-2,2,3,3-tetradeuteropropionic acid (TSP; δ0.00) and 0.1% (w/v) sodium azide. Sample aliquots were allocated to 96-well plates (and wells thereon) in a randomized design.

Each experiment was acquired on a Bruker DRX 600 MHz spectrometer (Rheinstetten, Germany) operating at 600 MHz (for ^1^H) using a 5 mm TXI flow-injection probe equipped with a *z*-gradient coil, at 300 K, at a spectral width of 12019 Hz, with 96 transients being collected with 8 dummy scans using 64k time domain data points. For both plasma and urine samples a standard 1D spectrum [RD−90°−3 µs−90°−*t*_m_−90°−acquire] with selective irradiation of the water resonance during the relaxation delay (RD, 2 s) and during the mixing time (*t*_m_, 0.1 s) was acquired. Additionally, for the plasma samples, a spin-echo (Carr-Purcell-Meiboom-Gill) spectrum [RD−90°−(τ/2−180°−τ/2)*n*−acquire] with a total echo time of 608 ms (*n* = 304, τ = 2000 µs) and a diffusion-edited spectrum made using a bipolar pulse-pair longitudinal eddy current delay pulse sequence with spoil gradients immediately following the 90° pulses after the bipolar gradient pulse pairs were acquired. Continuous wave irradiation was applied during the relaxation delay at the frequency of the water (or HOD) resonance. Eddy current recovery time (*T*_e_) was 5 ms, and the time interval between the bipolar gradients (|) was 0.5 ms. Further details may be found in [15], [26], [63].

### Data preprocessing and feature extraction (^1^H NMR)

Each of four data sets was passed independently through a semi-automated preprocessing pipeline: phasing, alignment, denoising, baseline correction, manual bin selection, normalization, quality control, peak extraction, and logarithmic transformation.

Spectra were phased using in-house software (NMRProc, T.M.D Ebbels and H.C. Keun, Imperial College London). All other data analysis was performed in R [64]. Spectra were zero-filled to 2^16^ points. Urine spectra were aligned to TSP, set at δ0.00; plasma spectra were aligned to formate, set at δ8.452 (peak centres were defined by the position of the local maximum).

The spectra were denoised in the frequency domain using wavelet-based methodology (a method similar to that described in [65]). For baseline correction, we initially fitted a constant baseline to each spectrum; however, visual inspection revealed that, for a number of spectra, the fit was better on one side of the water peak than on the other; natural variations in ionic strength resulting in altered phase of the residual water resonance may contribute to such an effect. Hence, a two-piece piecewise-constant baseline was fitted to and subtracted from each spectrum; specifically, the baseline on each side of the water peak was estimated by the 5th percentile of the spectral points in the corresponding interval (a robust estimator of baseline location).

We plotted each peak; for those peaks that visually displayed consistent presence across spectra, we manually created a bin and used the bin to extract the peak's data across all spectra. The datum extracted from a bin in a spectrum was the intensity of the highest local maximum (i.e. we used peak height as a proxy for peak area), or was coded as a missing value if no local maximum was present. We chose peak height to be the estimator of concentration as, in addition to its simplicity, it had relatively good robustness properties in the context of spectral artefacts (e.g. when a peak's location varied across spectra, or when neighbouring peaks overlapped within spectra). If the width (at half height) of a peak varies substantially across spectra, then peak height may be less precise than area at quantifying concentration. Plots of peaks did not reveal substantial peak-width variation in our data sets.

Only common peaks—present in at least 80% of spectra in their corresponding data set—were included in downstream statistical analysis, and only a peak's non-missing data were included at the statistical modelling stage. A missing datum, corresponding to there being no local maximum in the peak's ppm interval, typically occurred for one of two reasons: (a) the corresponding metabolite's concentration was too low to create a local maximum, or (b) a relatively large neighbouring peak overlapped the peak of interest (i.e. the missing concentration is censored, but not necessarily low). The omission of type (a) missing values from the analysis potentially decreased statistical power to detect mQTLs driving metabolite concentration variation at levels near or below the level of detection. The omission of type (b) missing values from the analysis increased the robustness of inference (and conserved power) in the face of artefactual effects of overlapping peaks. To illustrate, in Figure S7 we plotted the seven spectra (out of 432) with missing values for DMAp, and the four spectra (out of 432) with missing values for N-ACu. (There were no missing values for TMAu and BAIBu.) At the DMAp peak, missing data were representative of relatively low concentrations, approximately within the lowest quartile of observed concentrations (so we may have lost a small amount of power through missing-data handling). For N-ACu's missing data, the relevant peak's size was obscured by signal from an overlapping peak (missing values did not necessarily correspond to near-zero concentration).

Prior to model fitting, we discarded any peaks that were annotated to exogenous metabolites (of ibuprofen or acetaminophen), to a spike-in compound (TSP in urine, formate in plasma), or to urea (the area of which is affected by water peak saturation irradiation through chemical transfer of saturated protons). Across the three plasma data sets, 104 peaks were annotated to glucose; we discarded all but one representative glucose peak in each plasma data set.

The spectra were normalized using probabilistic quotient normalization [66]. The normalization was performed using data from the retained peaks only; spectra were normalized to a reference spectrum comprising median peak heights; missing values were excluded from the calculation of medians. After quality control, urine spectra were available for 142 MolTWIN participants and 67 MolOBB participants; plasma spectra were available for 140 MolTWIN participants and 68 MolOBB participants. A logarithmic transformation was applied to make the peak height distributions more symmetric–the entire spectrum-wide set of peak heights were collectively shifted and scaled to lie between zero and 100 and then transformed 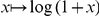.

### Genome-wide association scan (MolTWIN ^1^H NMR data)

We tested each metabolite peak in turn for association with 2,541,644 autosomal SNPs. For this stage we averaged and transformed the peak data as follows: (i) we averaged each subject's metabolite peak data across all biological and technical replicates; (ii) for robustness, we mapped the quantiles of the resulting inter-subject distribution to the quantiles of a standard Gaussian distribution. We denote the resulting data vector by 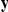. We fitted the following additive genetic model by ordinary least-squares regression at each SNP:
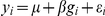
where 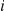 indexed subject; 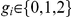 was the number of copies of the reference allele possessed by individual 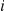; and 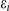 was the residual error term. At each SNP, we calculated the conventional *t*-statistic for the test of the null hypothesis 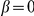. We then took the maximum absolute *t*-statistic observed across all SNPs tested, and this statistic, 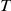, was the test statistic used for testing the null hypothesis, 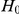: the metabolite's concentration was not associated with variation at any SNP in the genome-wide panel.

We characterized the (metabolite peak-specific) null distribution of 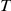 by permutation. For each of 5,000 permutations, we randomly reassigned the measured metabolite levels of each MZ pair to a different MZ pair, and randomly reassigned the measured metabolite levels of each DZ pair to a different DZ pair, yielding 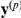 for the 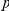
th permutation. Such a permutation crucially preserved the existing covariance structure on 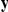 induced by polygenic genetic relatedness (identity-by-descent sharing) and common-environmental effects between twins, while breaking down any existing associations between 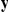 and identity-by-state variation at SNPs. For the 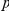
th permutation, we calculated *t*-statistics as before, quantifying the additive genetic association between 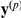 and the genotypes at each SNP. We then calculated the maximum absolute *t*-statistic across SNPs, yielding the 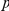
th draw from the null distribution, 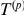.

For each metabolite, we rejected 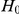 only if the observed test statistic exceeded all 5,000 draws from its null distribution, i.e. if 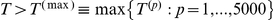. Such a procedure constrained (to be small) the family-wise error rate (FWER) for testing a single metabolite against genome-wide SNP variation. Specifically, (0, 0.0007) was an exact 95% confidence interval for the FWER, based on the observation that none of the 5,000 draws from the null distribution of 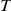 exceeded the observed statistic [67]. We concluded that our testing procedure controlled the false-positive probability for each metabolite's entire genome-wide scan to be less than 0.001.


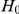
 was rejected for six of the 526 metabolite peaks tested. These six peaks redundantly represented four metabolites, listed in Table 2. For each metabolite, we examined the subset of SNPs that reached genome-wide significance (defined as those SNPs whose *t*-statistics exceeded, in absolute value, the metabolite's maximum null test statistic, 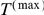; shown in Table S1). For each metabolite, the set of genome-wide significant SNPs co-localized to a single genomic region; we defined a metabolite's *hit region* to be the smallest contiguous region containing all genome-wide significant SNPs.

### Mixed-effects analysis of hit regions (MolTWIN ^1^H NMR data)

Underlying the observed data at a metabolite peak (i.e. across all spectra) was a complex correlation structure, induced by the sharing of alleles, individuals, and samples by different sample aliquots. In the follow-up analysis of hit regions we explicitly modelled this covariance structure while quantifying the metabolite's association with each local SNP in turn (i.e. with each SNP within 200 kb of the hit region and with MAF > 5%).

To deal with potential deviations from the Gaussian distributional assumptions, we mapped the quantiles of the empirical data distribution at each peak to the quantiles of a standard Gaussian distribution, yielding the transformed data vector, 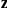. In contrast to the genome-wide analysis described in the previous section (based on the averaged data, 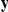), technical and biological replicates were not averaged for this analysis (instead, variation between replicates was retained in 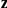 and modelled). We fitted the following mixed-effects model: 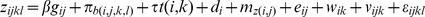
where twin pairs were indexed by 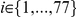, the twins within a pair were indexed by 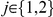, the visits of a twin pair were indexed by 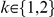, and the aliquots of a sample were indexed by 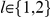. The ‘fixed effects’ in the model were 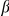, the 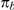, and 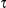. The additive effect of the SNP under consideration was modelled by 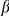, with 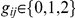 denoting the number of copies of the reference allele possessed by twin 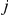 in pair 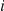. The parameters 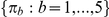 controlled for experimental inter-plate effects, with 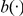 mapping spectra to plates. The parameter 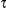 controlled for sampling time-related effects, with 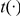 in the equation above mapping visits to sample-collection times (in 24-hour format; times were mostly 10 or 14). The other terms in the model were ‘random effects,’ which modelled the covariance structure across observations induced by familial 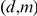, individual-environmental 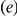, temporally dynamic 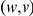, and non-biological 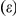 effects. Similarly to [68], there was one 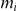 term for each MZ pair and two such terms, 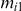 and 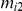, for each DZ pair (i.e. 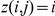 if 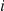 was an MZ pair, whilst 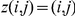 if 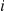 was a DZ pair). Each ‘random effect’ followed a zero-mean Gaussian distribution with its corresponding standard deviation from 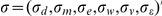 (e.g. the 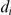 independently followed 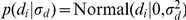).

The current paper's model induced a covariance structure on 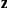 that was identical to that which is used in the standard methodology for modelling twin data (see, e.g., [68]–[70]). In the parameterization above, 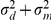 modelled the *familial* variance (i.e. the variance attributable to genetics and common environment), 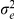 modelled the individual-environmental variance. Additionally, our model included variance parameters representing longitudinally unstable variation. These (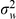 and 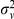) were referred to as the ‘common-visit’ and ‘individual-visit’ effects respectively, because they measured the component of phenotypic variation that fluctuated between visits, and which was shared and non-shared respectively between twins; the common-visit parameterization was included in the model because twins visited the clinic in pairs. Finally, there was a parameter 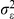 to model experimental variation. In the variance decompositions of the current paper (Figure 4 and Table 4), variances were expressed as proportions of the total biological variance, which was defined as 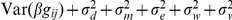, where 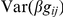 was the phenotypic variance explained by the corresponding mQTL SNP. The biological variance did not include the experimental variance, 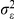, and was therefore appropriate for comparing the properties of molecular phenotypes across platforms when the level of experimental variation on the platforms was not of primary interest.

For each SNP within 200 kb of the hit region, we fitted the mixed-effects model both with and without the 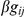 term. From these fitted models, we calculated the p-value for the test of the null hypothesis that 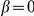, using 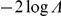 as a test statistic (where 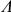 denotes the likelihood ratio), and employing its asymptotic null distribution (a chi-squared density with one degree of freedom). These p-values are displayed in the text, Figure 1, Figure 2, Figures S2 and S3, and Table 3, Tables S1 and S2A.

At the most strongly associated SNP, we went on to fit the model in a Bayesian framework, quantifying the precision of parameter estimates using posterior credible intervals. For this analysis we used directly the log-transformed metabolite concentrations, denoted by 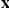 (see section on Data preprocessing and feature extraction). For priors, we specified Uniform densities on the standard deviation parameters in 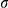 (as discussed in [71]):
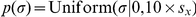
where 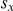 denotes the sample standard deviation of the data, 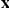. The prior on the ‘fixed effects’ vector, 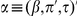, was a diffuse multivariate Gaussian distribution, with mean at the least squares estimates, 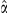, and diagonal covariance matrix with entries 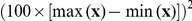. The results of fitting the model in a Bayesian framework are summarized in Table 4 and Figure 4.

### Replication of mQTL hits (MolOBB ^1^H NMR data)

For each of the mQTLs discovered in the MolTWIN cohort, we re-tested the association using only data from the MolOBB cohort. Specifically, we mapped the quantiles of the metabolite's concentration data to the corresponding quantiles of a standard Gaussian distribution; we then tested for an additive association with the corresponding SNP's genotype data, including age and gender as covariates in the linear model. Resulting p-values are shown in Table 3.

### Replication of Illig et al.'s mQTL hits (MolTWIN and MolOBB Biocrates data)

We used the concentration data directly as output from the Biocrates platform, and calculated metabolic traits from concentration ratios as in [14]. We removed individuals overlapping with the TwinsUK cohort used in [14], after which a total of 202 individuals were included in our Biocrates replication analysis (133 MolTWIN participants and 69 MolOBB participants). We fitted similar models to those specified in the *Materials and Methods* subsection ‘Mixed-effects analysis of hit regions,’ though now with fixed effects for genotype (number of copies of reference allele), plate, age, and gender. For each metabolic trait, the genotype data in the model was from the single corresponding mQTL SNP as reported in [14]. The results of the non-Bayesian analysis are shown in Table 5 and the results of the Bayesian analysis are in Table 4 and Figure 4.

### Data availability

The data underlying the current paper's analyses are available for download from an FTP server (host: svilpaste.mii.lu.lv; login: Moltwin_NMR; password: Moltwin_NMR1; path: /home/George/PLoS_Genetics_mQTL_data).

## Supporting Information

Figure S1 Peaks in urine ^1^H NMR spectra that are driven by mQTL variation. In the bottom panel we plotted 50 spectra over a subset of the ppm axis (note that, conventionally, the ppm axis is plotted increasing from right to left). The top panels are zoomed-in views of peaks from the three mQTL-driven urine metabolites of the current paper. The vertical scale of the bottom panel differs from the vertical scale shared by the top three panels.
(TIF)

Figure S2Hit region for DMAp. Top: location of genes, with rectangles denoting the position of exons. Middle: log-transformed p-values (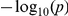) for the test of association of the metabolite's concentration with each SNP in the region. Bottom: LD between each pair of SNPs in the region, with the colour scale for 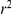 superimposed.(TIF)

Figure S3Hit region for BAIBu. Top: location of genes, with rectangles denoting the position of exons. Middle: log-transformed p-values (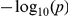) for the test of association of the metabolite's concentration with each SNP in the region. Bottom: LD between each pair of SNPs in the region, with the colour scale for 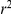 superimposed.(TIF)

Figure S4 Comparison of estimates of effect size of mQTL SNPs for metabolic traits measured on the Biocrates platform. Effect sizes are compared between Illig et al. (estimates are taken from Table 1 of [14]), and the current paper's replication of Illig et al.'s findings. The comparison is made using proportions of *total* phenotypic variance, because this was the measure of effect size used in [14]. Where applicable, the posterior distribution of effect size is represented as follows: the central tick in a box marks the posterior mean, the ends of a box mark the posterior quartiles, and the whiskers represent the central 95% credible interval (extending to the 2.5 and 97.5 posterior percentiles).
(TIF)

Figure S5Comparison of estimates of effect size of mQTL SNPs on metabolite concentrations measured by ^1^H NMR. Effect sizes are compared between the discovery stage (MolTWIN cohort) and the replication stage (MolOBB cohort). The MolTWIN estimates and credible intervals are as shown in Figure 4. The MolOBB estimates had to be calculated differently to the MolTWIN estimates because of the absence of technical replication in the MolOBB cohort study design. To calculate the MolOBB estimates, we first fitted a linear model with logarithmically transformed metabolite concentration, 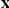, as the response variable, and with subjects' age, gender, and mQTL SNP genotype as explanatory variables—i.e. 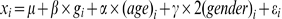, where 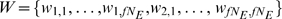 is the number of copies of the reference allele at the mQTL SNP carried by subject 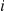. From the model fit, we estimated the proportion of *total* variance in metabolite concentration explained by the SNP using the ratio of sample variances: 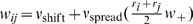. We then rescaled this to be the proportion of *biological* variation in metabolite concentration. This was achieved by dividing by 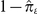, where 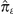 is the estimate (from the MolTWIN cohort) of the proportion of *total* variance in 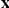 explained by experimental variation (see *Materials and Methods*).(TIF)

Figure S6 Distribution of the ratio of TMAOu concentration to the combined concentration of TMAOu and TMAu (includes both MolTWIN and MolOBB cohorts). Trimethylaminuria controls have relatively high values of TMAOu/(TMAOu + TMAu), typically greater than 0.8 [50], whilst values for cases are considerably lower (the two cases examined in [50] have values 0.11 and 0.22).
(TIF)

Figure S7 Plot of spectra for which mQTL-driven metabolites, labelled top, are determined as missing. Missing-peak spectra are plotted in black. For comparison, an arbitrarily selected set of 25 present-peak spectra is plotted in grey. Vertical green lines delimit the corresponding peak's bin (*Materials and Methods*).
(TIF)

Table S1Details of statistical association between each mQTL-driven metabolite and the SNPs within 200 kb of its hit region. Genomic locations are given in NCBI build 37 coordinates. Columns labelled ‘Beta,’ ‘S.E. Beta’ (S.E.  =  standard error) and ‘p-value’ (for the test of the null hypothesis that 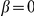) give details of the fit of the non-Bayesian mixed-effects model described in section ‘Mixed-effects analysis of hit regions (MolTWIN ^1^H NMR data)’ of *Materials and Methods*. The meaning of the column ‘Genome-Wide Significant’ is described in the final paragraph of the section ‘Genome-wide association scan (MolTWIN ^1^H NMR data)’ of *Materials and Methods*.(XLS)

Table S2 (A) Non-synonymous SNPs in LD with mQTL SNPs. (B) Corresponding residue changes and predicted functional effects of non-synonymous SNPs.
(DOC)

Table S3 Previously discovered eQTLs within 200 kb of mQTL hit regions.
(DOC)
