# Supplementary figures and images for: A Genome-Wide Metabolic QTL Analysis in Europeans Implicates Two Loci Shaped by Recent Positive Selection
Source: PLoS Genet. 2011 Sep 8;7(9):e1002270. doi: 10.1371/journal.pgen.1002270 (PMC3169529; doi:10.1371/journal.pgen.1002270)

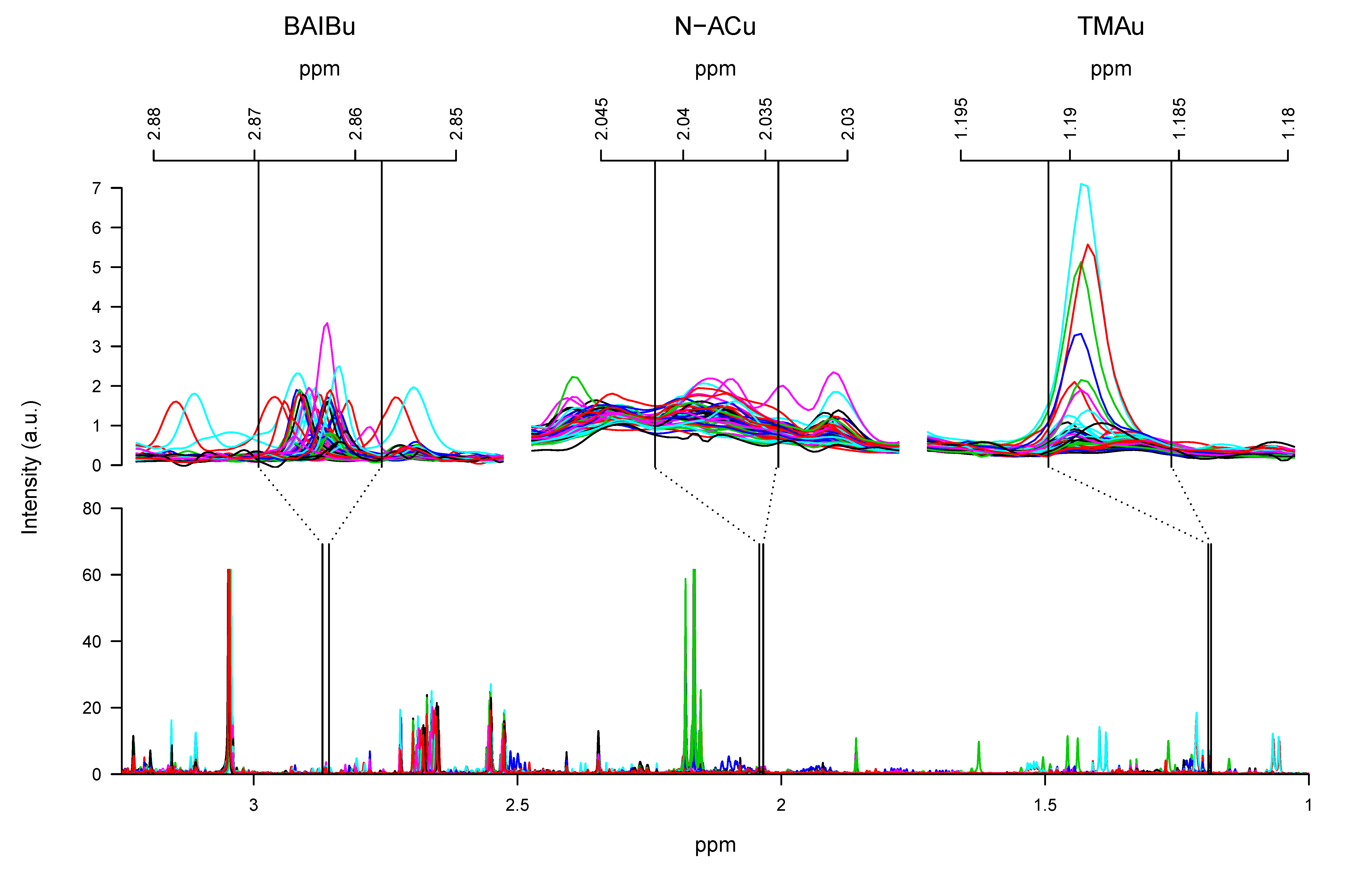

Supplement: Figure S1 — Peaks in urine 1H NMR spectra that are driven by mQTL variation. In the bottom panel we plotted 50 spectra over a subset of the ppm axis (note that, conventionally, the ppm axis is plotted increasing from right to left). The top panels are zoomed-in views of peaks from the three mQTL-driven urine metabolites of the current paper. The vertical scale of the bottom panel differs from the vertical scale shared by the top three panels. (TIF) [file pgen.1002270.s001.tif]

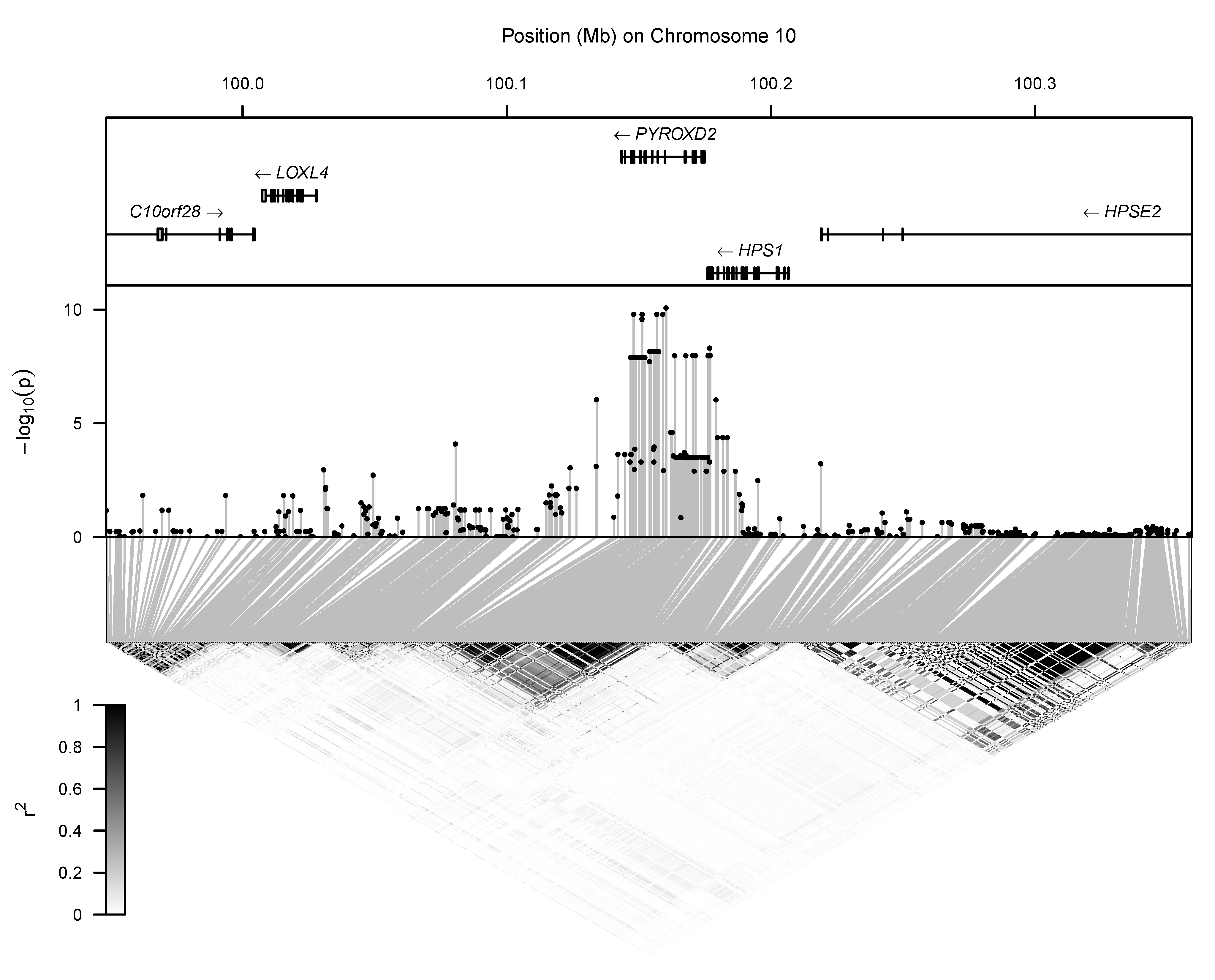

Supplement: Figure S2 — Hit region for DMAp. Top: location of genes, with rectangles denoting the position of exons. Middle: log-transformed p-values () for the test of association of the metabolite's concentration with each SNP in the region. Bottom: LD between each pair of SNPs in the region, with the colour scale for superimposed. (TIF) [file pgen.1002270.s002.tif]

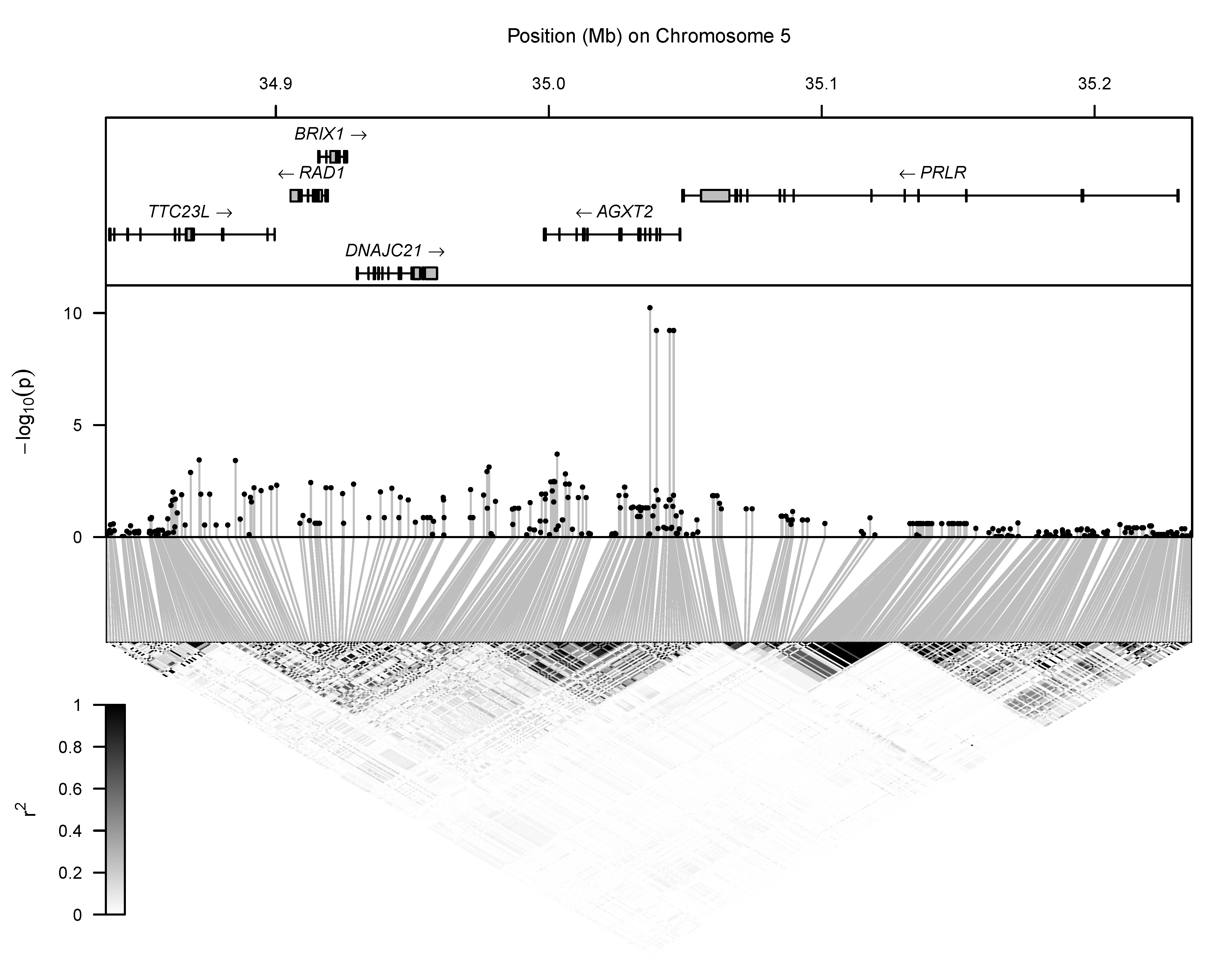

Supplement: Figure S3 — Hit region for BAIBu. Top: location of genes, with rectangles denoting the position of exons. Middle: log-transformed p-values () for the test of association of the metabolite's concentration with each SNP in the region. Bottom: LD between each pair of SNPs in the region, with the colour scale for superimposed. (TIF) [file pgen.1002270.s003.tif]

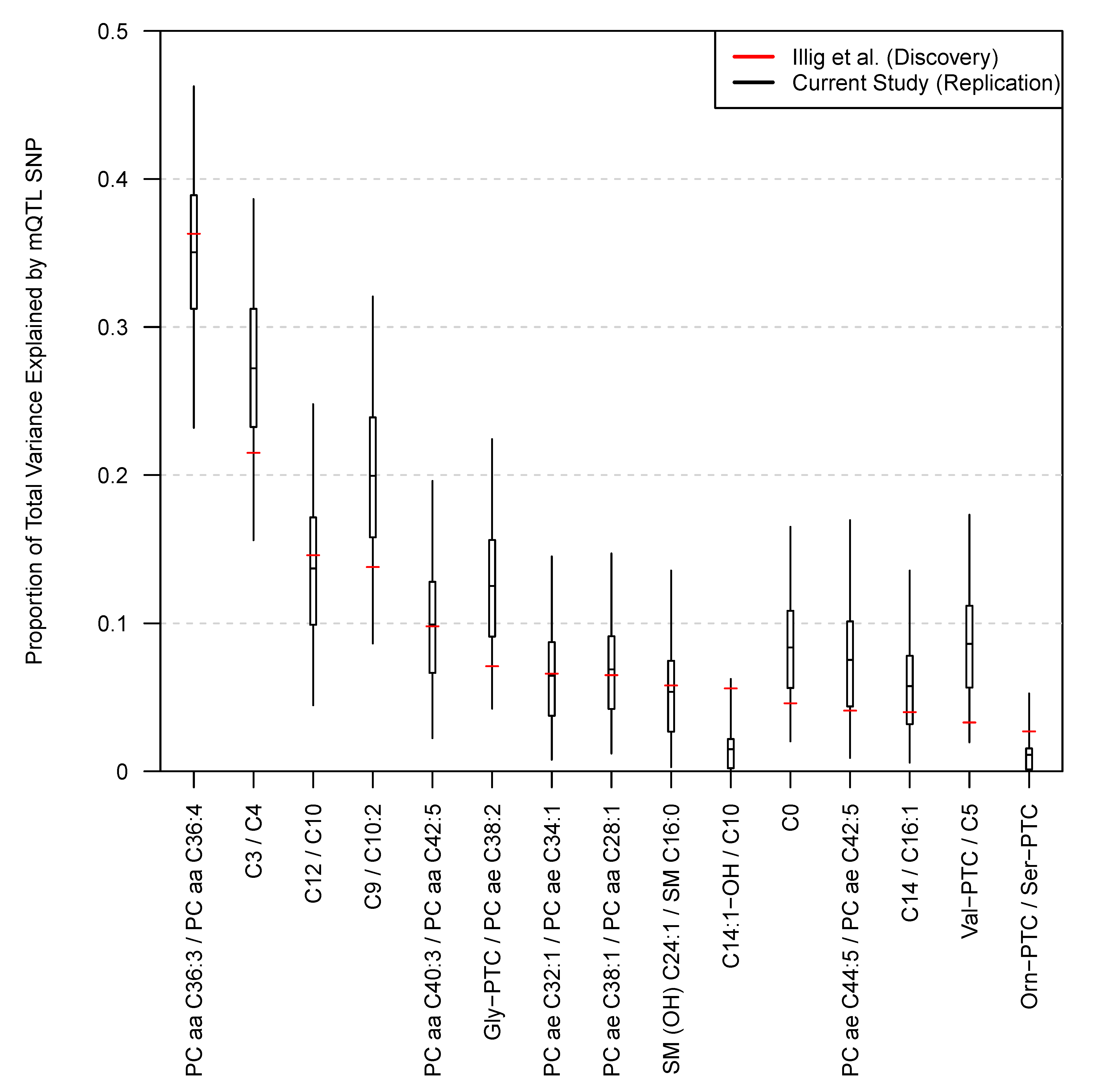

Supplement: Figure S4 — Comparison of estimates of effect size of mQTL SNPs for metabolic traits measured on the Biocrates platform. Effect sizes are compared between Illig et al. (estimates are taken from Table 1 of [14]), and the current paper's replication of Illig et al.'s findings. The comparison is made using proportions of total phenotypic variance, because this was the measure of effect size used in [14]. Where applicable, the posterior distribution of effect size is represented as follows: the central tick in a box marks the posterior mean, the ends of a box mark the posterior quartiles, and the whiskers represent the central 95% credible interval (extending to the 2.5 and 97.5 posterior percentiles). (TIF) [file pgen.1002270.s004.tif]

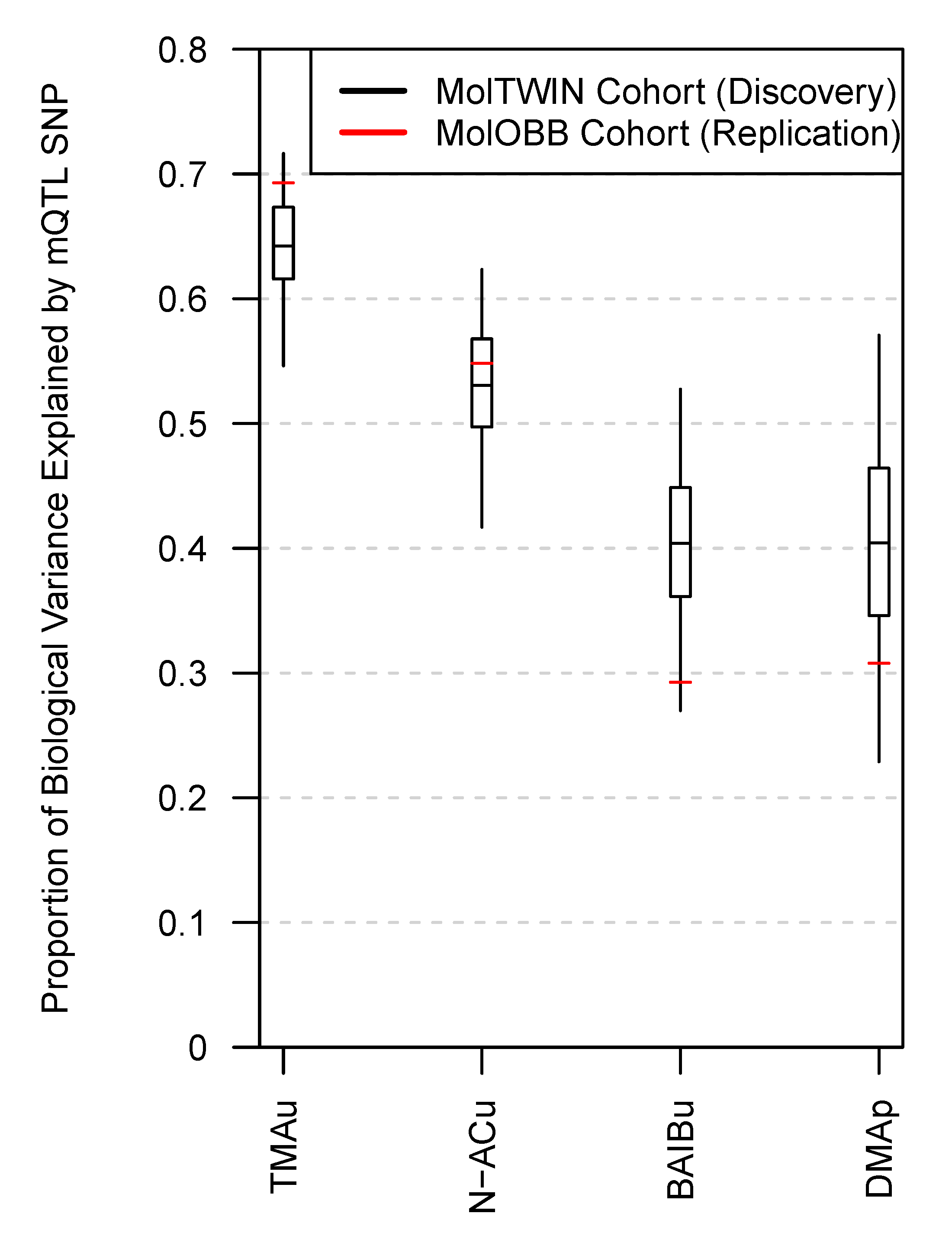

Supplement: Figure S5 — Comparison of estimates of effect size of mQTL SNPs on metabolite concentrations measured by 1H NMR. Effect sizes are compared between the discovery stage (MolTWIN cohort) and the replication stage (MolOBB cohort). The MolTWIN estimates and credible intervals are as shown in Figure 4. The MolOBB estimates had to be calculated differently to the MolTWIN estimates because of the absence of technical replication in the MolOBB cohort study design. To calculate the MolOBB estimates, we first fitted a linear model with logarithmically transformed metabolite concentration, , as the response variable, and with subjects' age, gender, and mQTL SNP genotype as explanatory variables—i.e. , where is the number of copies of the reference allele at the mQTL SNP carried by subject . From the model fit, we estimated the proportion of total variance in metabolite concentration explained by the SNP using the ratio of sample variances: . We then rescaled this to be the proportion of biological variation in metabolite concentration. This was achieved by dividing by , where is the estimate (from the MolTWIN cohort) of the proportion of total variance in explained by experimental variation (see Materials and Methods ). (TIF) [file pgen.1002270.s005.tif]

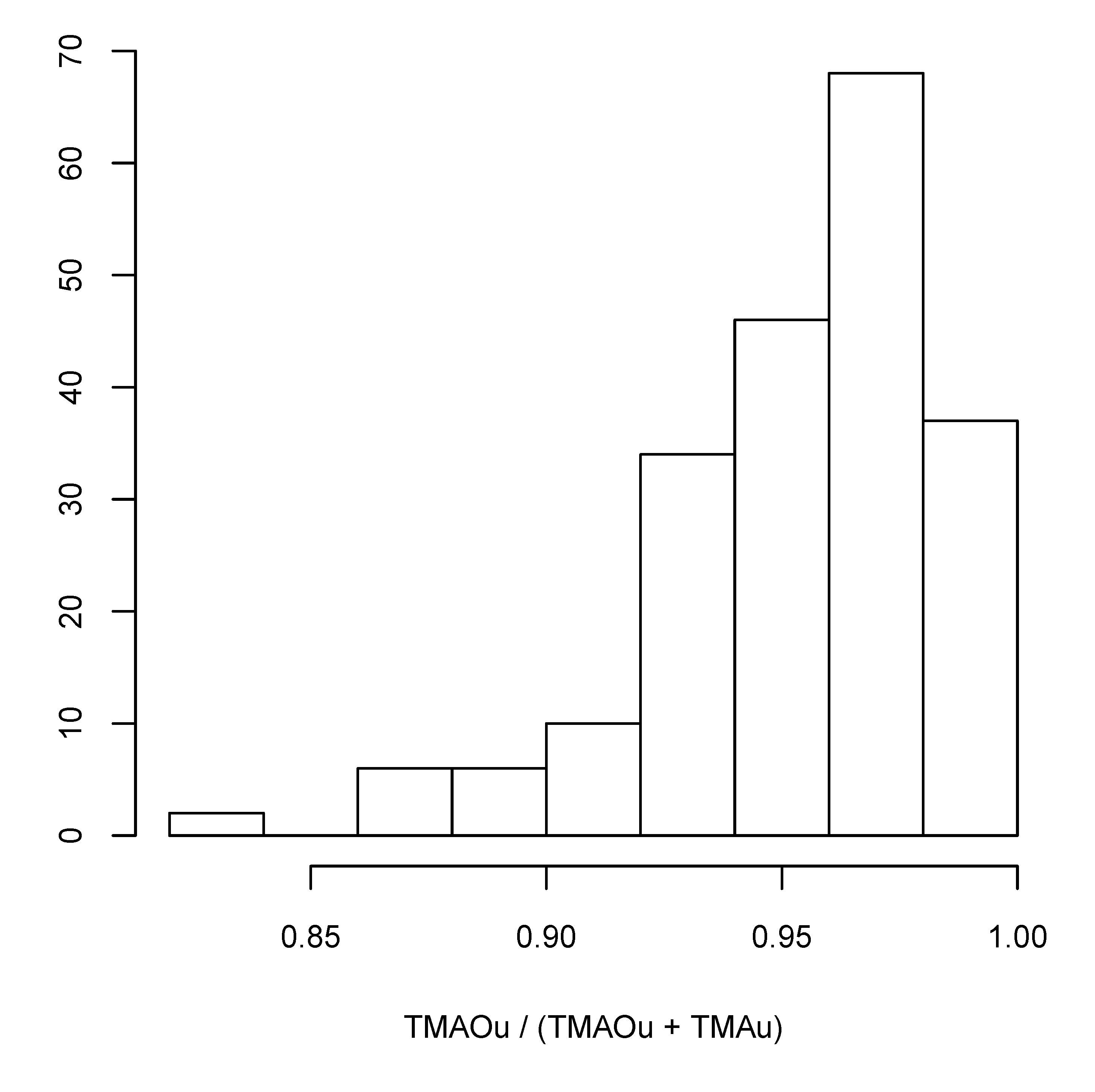

Supplement: Figure S6 — Distribution of the ratio of TMAOu concentration to the combined concentration of TMAOu and TMAu (includes both MolTWIN and MolOBB cohorts). Trimethylaminuria controls have relatively high values of TMAOu/(TMAOu + TMAu), typically greater than 0.8 [50], whilst values for cases are considerably lower (the two cases examined in [50] have values 0.11 and 0.22). (TIF) [file pgen.1002270.s006.tif]
