# Supplementary material for: A Genome-Wide Metabolic QTL Analysis in Europeans Implicates Two Loci Shaped by Recent Positive Selection
Source: PLoS Genet. 2011 Sep 8;7(9):e1002270. doi: 10.1371/journal.pgen.1002270 (PMC3169529; doi:10.1371/journal.pgen.1002270)
Supplement: Table S2 — (A) Non-synonymous SNPs in LD with mQTL SNPs. (B) Corresponding residue changes and predicted functional effects of non-synonymous SNPs. (DOC) [file pgen.1002270.s009.doc]

Table S2A. Non-synonymous SNPs in LD with mQTL SNPs

|  |  |  | Non-Synonymous SNP | | |  | Strongest-Effect SNP | | |
| --- | --- | --- | --- | --- | --- | --- | --- | --- | --- |
| ID | Gene | Chr | rs ID | Position | p-value[[1]](#footnote-2) | Inter-SNP LD[[2]](#footnote-3) | rs ID | Position | p-value |
| TMAu | PYROXD2 | 10 | rs2147896 | 100148176 | 2.8E-23 | 1.00 | rs7072216 | 100156853 | 2.8E-23 |
| TMAu | PYROXD2 | 10 | rs2296441 | 100144782 | 1.4E-08 | 0.71 | rs7072216 | 100156853 | 2.8E-23 |
| N-ACu | NAT8 | 2 | rs13538 | 73868328 | 9.0E-18 | 1.00 | rs9309473 | 73743982 | 4.1E-19 |
| BAIBu | AGXT2 | 5 | rs37369 | 35037115 | 5.9E-11 | 1.00 | rs37369 | 35037115 | 5.9E-11 |
| BAIBu | AGXT2 | 5 | rs37370 | 35039486 | 6.1E-10 | 0.74 | rs37369 | 35037115 | 5.9E-11 |
| DMAp | PYROXD2 | 10 | rs2147896 | 100148176 | 1.6E-10 | 0.92 | rs6584194 | 100160399 | 8.6E-11 |

Table S2B. Corresponding residue changes and predicted functional effects of non-synonymous SNPs

| ID(s) | Gene | Transcript ID | Non-Syn. SNP | Allele Change | Residue Change | Effect Direc.[[3]](#footnote-4) | SIFT[[4]](#footnote-5) | PolyPhen[[5]](#footnote-6) | PhyloP[[6]](#footnote-7) |
| --- | --- | --- | --- | --- | --- | --- | --- | --- | --- |
| TMAu, DMAp | PYROXD2 | ENST00000370575 | rs2147896 | G1431A | T461M | + | benign | tolerated | 1.07 |
| TMAu, DMAp | PYROXD2 | ENST00000370575 | rs2296441 | C1646T | A533T | - | benign | tolerated | 2.39 |
| N-ACu | NAT8 | ENST00000272425 | rs13538 | A578G | F143S | + | benign | tolerated | -2.63 |
| BAIBu | AGXT2 | ENST00000231420 | rs37369 | C619T | V140I | + | benign | tolerated | -0.22 |
| BAIBu | AGXT2 | ENST00000231420 | rs37370 | T506C | N102S | + | benign | tolerated | 0.96 |

Table S2 References

1. Kumar P, Henikoff S, Ng P (2009) Predicting the effects of coding non-synonymous variants on protein function using the SIFT algorithm. Nature protocols 4: 1073-1081.

2. Ramensky V, Bork P, Sunyaev S (2002) Human non-synonymous SNPs: server and survey. Nucleic Acids Res 30: 3894-3900.

3. Pollard K, Hubisz M, Rosenbloom K, Siepel A (2010) Detection of nonneutral substitution rates on mammalian phylogenies. Genome Res 20: 110-121.

4. Kuhn RM, Karolchik D, Zweig AS, Wang T, Smith KE, et al. (2009) The UCSC Genome Browser Database: update 2009. Nucleic Acids Res 37: D755-761.

1. Only non-synonymous SNPs with association p-values < 10^-7^  (arbitrary threshold) are included in the table. [↑](#footnote-ref-2)
2. Measure of LD (r^2^) in the HapMap-CEU population [1] between the non-synonymous SNP and the SNP with strongest effect at the mQTL [↑](#footnote-ref-3)
3. The observed directionality of the effect of the 'Allele Change' on the concentration of the specified metabolites ('ID(s)') [↑](#footnote-ref-4)
4. SIFT prediction [1] [↑](#footnote-ref-5)
5. PolyPhen prediction [2] [↑](#footnote-ref-6)
6. Placental mammal base-wise conservation by PhyloP [3,4]. PhyloP scores are calculated as the abs(log_10_(p-value)) for testing the null hypothesis of neutral evolution, and are then multiplied by -1 to indicate faster-than-expected evolution, or by +1 to indicate conservation. [↑](#footnote-ref-7)
