# Supplementary material for: A Genome-Wide Metabolic QTL Analysis in Europeans Implicates Two Loci Shaped by Recent Positive Selection
Source: PLoS Genet. 2011 Sep 8;7(9):e1002270. doi: 10.1371/journal.pgen.1002270 (PMC3169529; doi:10.1371/journal.pgen.1002270)
Supplement: Table S3 — Previously discovered eQTLs within 200 kb of mQTL hit regions. (DOC) [file pgen.1002270.s010.doc]

Table S3. Previously discovered eQTLs within 200kb of mQTL hit regions.

| Metabolite ID | mQTL SNP | mQTL-eQTL LD[[1]](#footnote-2) | eQTL SNP[[2]](#footnote-3) | eQTL Paper | eQTL Sample | eQTL Gene |
| --- | --- | --- | --- | --- | --- | --- |
| TMAu | rs2296438 | 1.00 | rs2147897 | Dimas et al. (2009) | Fibroblasts | C10orf33 |
| TMAu | rs11189600 | 1.00 | rs11189600 | Schadt et al. (2008) | Liver | HPS1 |
| TMAu | rs7896828 | 0.76 | rs2147901 | Schadt et al. (2008) | Liver | C10orf33 |
| TMAu | rs2274247 | 0.10 | rs1061134 | Schadt et al. (2008) | Liver | C5orf16 |
| TMAu | rs2274247 | 0.10 | rs1061134 | Schadt et al. (2008) | Liver | ENST00000300165 |
| TMAu | rs2274247 | 0.10 | rs1061134 | Schadt et al. (2008) | Liver | hCT2283696 |
| TMAu | rs2274247 | 0.10 | rs1061134 | Schadt et al. (2008) | Liver | IL16 |
| TMAu | rs2274247 | 0.10 | rs1061134 | Schadt et al. (2008) | Liver | ZNF780B |
| N-ACu | rs10496191 | 1.00 | rs10496191 | Montgomery et al. (2010) | LCLs | SMYD5 |
| N-ACu | rs11689392 | 1.00 | rs6710438 | Myers et al. (2007) | Cortex from control brain | ALMS1 |
| N-ACu | rs6546860 | 0.91 | rs1589576 | Schadt et al. (2008) | Liver | ALMS1 |
| N-ACu | rs6546860 | 0.91 | rs1589576 | Schadt et al. (2008) | Liver | STAMBP |
| N-ACu | rs6546849 | 0.76 | rs2567603 | Montgomery et al. (2010) | LCLs | AUP1 |
| N-ACu | rs6546852 | 0.59 | rs1052161 | Montgomery et al. (2010) | LCLs | DGUOK |
| N-ACu | rs6546849 | 0.39 | rs17350188 | Schadt et al. (2008) | Liver | TPRKB |
| N-ACu | rs7598396 | 0.30 | rs3813230 | Montgomery et al. (2010) | LCLs | TTC31 |
| N-ACu | rs7598396 | 0.30 | rs3813230 | Montgomery et al. (2010) | LCLs | WDR54 |
| N-ACu | rs6546849 | 0.23 | rs1474335 | Montgomery et al. (2010) | LCLs | TET3 |
| BAIBu | rs37369 | 0.10 | rs12519332 | Montgomery et al. (2010) | LCLs | DNAJC21 |
| BAIBu | rs37369 | 0.06 | rs10521021 | Schadt et al. (2008) | Liver | AGXT2 |
| DMAp | rs4539242 | 1.00 | rs2147897 | Dimas et al. (2009) | Fibroblasts | C10orf33 |
| DMAp | rs4539242 | 0.77 | rs11189600 | Schadt et al. (2008) | Liver | HPS1 |
| DMAp | rs2147896 | 0.65 | rs2147901 | Schadt et al. (2008) | Liver | C10orf33 |
| DMAp | rs4539242 | 0.09 | rs1061134 | Schadt et al. (2008) | Liver | C5orf16 |
| DMAp | rs4539242 | 0.09 | rs1061134 | Schadt et al. (2008) | Liver | ENST00000300165 |
| DMAp | rs4539242 | 0.09 | rs1061134 | Schadt et al. (2008) | Liver | hCT2283696 |
| DMAp | rs4539242 | 0.09 | rs1061134 | Schadt et al. (2008) | Liver | IL16 |
| DMAp | rs4539242 | 0.09 | rs1061134 | Schadt et al. (2008) | Liver | ZNF780B |

1. For each eQTL, we calculated r^2^ (CEU HapMap 3 data [29]) between the eQTL and each mQTL SNP in the hit region that reached genome-wide significance for association with the respective metabolite's concentration. The table shows the eQTL-mQTL pair with maximum r^2^. [↑](#footnote-ref-2)
2. We catalogued known eQTL SNPs within 200kb of each metabolite's hit region using a publicly available eQTL browser (http://eqtl.uchicago.edu/cgi-bin/gbrowse/eqtl/) [↑](#footnote-ref-3)
